# Sex Differences in Cerebral Small Vessel Disease: A Systematic Review and Meta-Analysis

**DOI:** 10.3389/fneur.2021.756887

**Published:** 2021-10-28

**Authors:** Lorena Jiménez-Sánchez, Olivia K. L. Hamilton, Una Clancy, Ellen V. Backhouse, Catriona R. Stewart, Michael S. Stringer, Fergus N. Doubal, Joanna M. Wardlaw

**Affiliations:** ^1^Translational Neuroscience PhD Programme, Centre for Clinical Brain Sciences, University of Edinburgh, Edinburgh, United Kingdom; ^2^Edinburgh Dementia Research Centre in the UK Dementia Research Institute, Edinburgh, United Kingdom; ^3^Centre for Clinical Brain Sciences, University of Edinburgh, Edinburgh, United Kingdom; ^4^Lothian Birth Cohorts, University of Edinburgh, Edinburgh, United Kingdom

**Keywords:** cerebral small vessel disease (SVD), sex differences, cerebral autosomal dominant arteriopathy with subcortical infarcts and leukoencephalopathy (CADASIL), lacunar stroke, vascular dementia (VaD)

## Abstract

**Background:** Cerebral small vessel disease (SVD) is a common cause of stroke, mild cognitive impairment, dementia and physical impairments. Differences in SVD incidence or severity between males and females are unknown. We assessed sex differences in SVD by assessing the male-to-female ratio (M:F) of recruited participants and incidence of SVD, risk factor presence, distribution, and severity of SVD features.

**Methods:** We assessed four recent systematic reviews on SVD and performed a supplementary search of MEDLINE to identify studies reporting M:F ratio in covert, stroke, or cognitive SVD presentations (registered protocol: CRD42020193995). We meta-analyzed differences in sex ratios across time, countries, SVD severity and presentations, age and risk factors for SVD.

**Results:** Amongst 123 relevant studies (*n* = 36,910 participants) including 53 community-based, 67 hospital-based and three mixed studies published between 1989 and 2020, more males were recruited in hospital-based than in community-based studies [M:F = 1.16 (0.70) vs. M:F = 0.79 (0.35), respectively; *p* < 0.001]. More males had moderate to severe SVD [M:F = 1.08 (0.81) vs. M:F = 0.82 (0.47) in healthy to mild SVD; *p* < 0.001], and stroke presentations where M:F was 1.67 (0.53). M:F did not differ for recent (2015–2020) vs. pre-2015 publications, by geographical region, or age. There were insufficient sex-stratified data to explore M:F and risk factors for SVD.

**Conclusions:** Our results highlight differences in male-to-female ratios in SVD severity and amongst those presenting with stroke that have important clinical and translational implications. Future SVD research should report participant demographics, risk factors and outcomes separately for males and females.

**Systematic Review Registration:** [PROSPERO], identifier [CRD42020193995].

## Introduction

Cerebral small-vessel disease (SVD) is a disorder of the brain small penetrating blood vessels leading to white and deep gray matter damage, and is a major cause of stroke (1) and dementia (2).

Sex differences occur in many vascular diseases (3), and they can also be expected in the context of SVD. SVD is most commonly sporadic, although there are rare familial types, like cerebral autosomal dominant arteriopathy with subcortical infarcts and leukoencephalopathy (CADASIL), which although not sex-linked, seems to affect males more severely than females (4). Covert SVD is common in older persons, where differences in male:female incidence or severity have not been assessed. Several studies of stroke of any type have recruited more males than females and reported a higher age-adjusted incidence in males, but higher severity in females. However, as reviewed elsewhere, it is unclear whether these conclusions reflect underlying sex-specific biological differences, recruitment bias, or other factors (5). On average, females are older than males at stroke onset, more likely to live alone and have more severe baseline deficits (6), which could explain their increased pre-hospital delay, and their higher severity in first-ever acute stroke (7). These factors can affect females' eligibility for stroke research studies, with a bias toward recruitment of milder strokes, and for stroke treatment, as females are less likely to be treated with IV thrombolysis than males (8). Interestingly, females were more likely to refuse participation in stroke clinical trials than males independently of their age (9).

Globally, females tend to live longer than males but there is a lack of sex and gender-stratified data in aging research (10) that may impede more personalized care in older populations; especially when biological factors, treatments, or social disparities may differ between sexes (11). Understanding male:female differences in incidence or severity of disease, particularly of common diseases like SVD, is now a World Health Organization imperative.

We aimed to explore if there are sex differences in covert or clinical presentations of SVD by assessing the sex ratio of participants with clinical or radiological evidence of SVD recruited to a range of studies, and whether any difference could be explained by male:female differences in risk factors or the severity of SVD features.

## Methods

We followed the Preferred Reported Items for Systematic Reviews and Meta-Analysis (PRISMA) guidelines and registered the protocol on PROSPERO on July 2, 2020 (CRD42020193995) (12).

### Data Sources and Search Strategy

We identified relevant studies in two ways.

First, we examined studies that had been included in four recent published systematic reviews of different aspects of SVD, whose search terms were similar as for the current work, and identified studies that met our current inclusion criteria (see below). The four systematic reviews addressed (a) early life risk factors for SVD (13), (b) cognitive dysfunction in SVD (14), (c) neuropsychiatric and cognitive symptoms in SVD (15), and (d) cerebral blood flow in SVD (16). We used the four published systematic reviews as a highly efficient way to access a large relevant literature, a practice which is now endorsed as part of the drive toward reducing research waste, improving efficiency, and best practice in evidence synthesis (17, 18). These systematic reviews had already been thoroughly screened and quality assessed, had each been conducted according to PRISMA guidelines, used relevant search terms, performed study quality assessment, had undergone peer review, and been published (Table 1) (13–16). Each systematic review had assessed a very large literature on a different aspect of SVD, enabling us to assess a very large number of relevant studies as efficiently as possible.

**Table 1:** Systematic reviews.

**Study** (13–16) **(primary author, year)**	**Title**	**Identified studies**	**Included studies**	**Total number of included participants in each review**
Backhouse, 2017	Early life risk factors for cerebrovascular disease.	19,180	29	23,356
Clancy, 2020	Neuropsychiatric symptoms associated with cerebral small vessel disease: a systematic review and meta-analysis.	7,119	81	21,730
Hamilton, 2020	Cognitive impairment in sporadic cerebral small vessel disease: a systematic review and meta-analysis.	8,562	69	6,908
Stewart, 2020	Associations between white matter hyperintensity burden, cerebral blood flow and transit time in small vessel disease: an updated meta-analysis.	783	30	3,396

*Identified studies refer to those found by search after duplicates were removed. Included studies refer to those examined for data extraction. The number of included studies of each systematic review do not include duplicated studies or populations*.

Second, we designed an independent search to supplement all the studies collected from the four systematic reviews with recent publications. We used a search strategy modified from a published protocol (14) to identify studies including participants with clinical (stroke or cognitive presentations) or non-clinical presentations of sporadic or monogenic SVD (e.g., CADASIL). Stroke presentations included lacunar syndromes with corresponding small subcortical infarct on neuroimaging, or that excluded other causes of symptoms. Cognitive presentations included vascular cognitive impairment, either vascular mild cognitive impairment—VaMCI—or vascular dementia—VaD. Non-clinical presentations included radiological evidence of SVD—e.g., white matter hyperintensities (WMH), lacunes of presumed vascular origin, small subcortical infarcts or cerebral microbleeds (CMBs) on brain magnetic resonance imaging (MRI) (19)—in the absence of clinical diagnosis (generally in community-dwelling populations), i.e., “covert” SVD. We aimed to explore trends across time by comparing recent and previously published studies (see *Results, Trends across time*) and decided to use January, 1, 2015 as the starting publication date for recent studies. Thus, January, 1, 2015 was the first date used for the independent search. We searched MEDLINE through OVID for human studies published in English or Spanish from January, 1, 2015 to May, 26, 2020 as follows: Cerebral Small Vessel Diseases/ OR (small vessel disease or small vessel-disease or CSVD or SVD).ti.ab. OR stroke,Lacunar/ OR [(lesion^*^ or hyperinten^*^) adj3 white matter].ti.ab. OR Leukoaraiosis/ OR lacune^*^.ti.ab. OR [(lacun^*^ or subcort^*^ or ischemi^*^ or ischaemi^*^ or silent or microscopic) adj3 lesion^*^).ti,ab.]. Since the used search strategy was modified from one of these systematic reviews' protocol, only the most recent 150 journal articles among the 4,871 filtered results were examined to avoid retrieving duplicated studies that were already present in the database. The electronic search was carried out on May, 26, 2020.

### Study Selection

We included cross-sectional and longitudinal studies published in English or Spanish that considered clinical diagnosis of SVD, radiological markers for SVD or studies reporting on patients with stroke that provided data according to stroke subtype (cortical or lacunar stroke). We excluded studies that did not report proportions of males and females or stroke subtype in the case of studies in stroke, review papers other than the included systematic reviews, editorials, communications, case reports, case series and conference abstracts, studies about other neurodegenerative conditions (e.g., Parkinson's disease, Alzheimer's Disease, non-vascular, or mixed dementia), inflammatory disorders (e.g., encephalitis/meningitis/vasculitis), single-sex populations (e.g., pregnancy studies), and genetic-based studies that only recruited from families. To avoid possible confounding factors related to large vessel disease, studies that recruited participants based on cardiovascular events (e.g., heart failure) and diffuse cardiovascular disease (e.g., atherosclerosis) were also excluded. The population of interest was patients presenting with stroke-related SVD (lacunar stroke), cognitive impairment found to have radiological features of SVD on neuroimaging, or participants with no clinical presentation found to have radiological features of SVD on neuroimaging (covert SVD). SVD radiological features included WMH, lacunes, small subcortical infarcts, CMBs, silent brain infarcts, or prior hemorrhage.

Where more than one study presented data on the same population, the study considering the most information about SVD clinical diagnosis, radiological markers, or risk factors for SVD was selected.

### Data Extraction

Screening, full-text review, study selection and data extraction were independently carried out by five authors (LJ-S, OKLH, EVB, UC, and CRS). Studies included in the published systematic reviews had already been assessed by two researchers. Studies identified in the new literature search were assessed by one researcher and cross-checked with another researcher in the case of uncertainty about inclusion.

We extracted data on the primary author, date of publication, country of recruited participants, study type (cross-sectional or longitudinal), clinical or non-clinical presentation of participants (including lacunar or subcortical stroke or hemorrhagic forms of SVD, subjective memory or cognitive complaints, VaMCI, VaD, or covert SVD), number of subjects, total sex ratio of participants, mean age of participants and sex-stratified mean age of participants, stratified sex ratio by clinical diagnosis of SVD, radiological features of SVD (presence and severity of WMH, lacunes, small subcortical infarcts, CMBs, silent brain infarcts or prior hemorrhage) or SVD score if provided. We calculated mean ages if not reported and data were available. Hypertension and current or ever-smoking data were recorded if available, since these are key modifiable risk factors known to worsen SVD (20), and calculated sex-stratified percentages of hypertension and smoking. An initial screening of papers indicated that there would be few sex-stratified data to explore other risk factors. We only extracted baseline data in longitudinal studies.

### Statistical Analysis

We performed all analyses and generated plots using R (version 3.2.3) (21). We calculated sex ratios of study participants or SVD groups of all the included studies and compared sex ratio per type of SVD presentation. Our principal summary measure was the median sex ratio per study setting, SVD presentation and severity.

Since recruitment can be affected by different factors across different settings, we classified studies into community-based, hospital-based, or mixed (where participants were recruited from both community and hospitals). To investigate whether differences in sex ratios were influenced by study size, we calculated a new variable: Δ sex ratio = |a constant of the global population sex ratio (22) – sex ratio of each study|. We log-transformed sizes of recruited populations due to their skewed distribution and then assessed the correlation of Δ sex ratio with the log-transformed size of the recruited populations per study population type.

To explore trends across time and countries, we classified studies by year of publication and country of recruited participants, respectively. To explore trends across severity and presentations of SVD, we then classified participants into healthy to mild SVD (mild covert SVD) vs. moderate to severe SVD (stroke presentations, cognitive presentations, moderate to severe covert SVD and genetic SVD; detailed in Table 2).

**Table 2:** Study classification by SVD severity and presentation.

**Group**	**Description**
Healthy to mild SVD	According to the definitions used in the original articles from which data was extracted: those defined as neurologically, functionally or cognitively healthy, community-dwelling individuals or participants with mild covert SVD (no clinical presentation with radiological features of SVD originally described as “mild”: deep or periventricular WMH, white matter lesions, vascular white matter disease, lacunes, leukoaraiosis, CMBs, silent brain infarcts, or ICH).
Moderate to severe SVD	According to the definitions used in the original articles from which data was extracted: those with moderate or severe clinical or non-clinical presentations of SVD. This group included stroke presentations, cognitive presentations, moderate to severe covert SVD and genetic SVD.
Stroke presentations	Those first presenting with a lacunar or subcortical stroke or lacunar syndrome. Since cerebrovascular events can precede cognitive impairment, participants with both stroke and cognitive presentations of SVD (e.g., participants with lacunar stroke who also presented with VaD) were considered part of the stroke presentations group rather than the cognitive presentations group.
Cognitive presentations	Those presenting with self-reported and/or diagnosed cognitive impairment (subjective cognitive/memory complaints, subjective cognitive decline, VaMCI, VaD, subcortical ischemic vascular dementia or multi-infarct dementia).
Moderate to severe covert SVD	Those with no clinical presentation found to have radiological features of SVD originally described as “moderate” or “severe” (deep or periventricular WMH, white matter lesions vascular white matter disease, lacunes, leukoaraiosis, CMBs, silent brain infarcts, or ICH).
Genetic SVD	CADASIL

*CADASIL, cerebral autosomal dominant arteriopathy with subcortical infarcts and leukoencephalopathy; CMB, cerebral microbleeds; ICH, intracerebral hemorrhage; SVD, cerebral small vessel disease; VaD, vascular dementia; VaMCI, vascular mild cognitive impairment; WMH, white matter hyperintensities*.

For quantitative analyses, we used Shapiro-Wilk tests to check for data normality. Sex ratio and sex-stratified data were not normally distributed, so we used non-parametric statistical tests. We used the Mann-Whitney-Wilcoxon test to explore comparisons between two groups and the Kruskal-Wallis test to explore comparisons between more than two groups. If the result of the Kruskal-Wallis test was significant, we further analyzed data by pairwise Mann-Whitney-Wilcoxon followed by Bonferroni *post-hoc* correction. We assessed correlations using Spearman's rank correlation coefficient. In text, we present data as median (interquartile range, IQR) with significance threshold set at *p* < 0.05.

### Study Quality Assessment

We performed a quality assessment of all the studies identified through the systematic reviews and the supplementary search of the recent literature as previously (14), rated on a scale from 0 to 8 according to STROBE guidelines, and calculated the median and IQR of the quality score. To check sensitivity, we re-ran the meta-analyses excluding studies with quality scores lower than the median quality score of all included studies.

### Risk of Bias Assessment

Bias refers to factors that can systematically affect the observations and conclusions of the study, making them differ from the truth (23). Relevant biases for this systematic review could be explored in studies that compared SVD incidence, severity, or risk factors in strata by sex. However, since very few studies have been published specifically on male-to-female ratios in SVD, and none (to our knowledge) aimed specifically to assess male:female differences in SVD, risk of bias was not assessed in this study.

## Results

**Figure 1:**
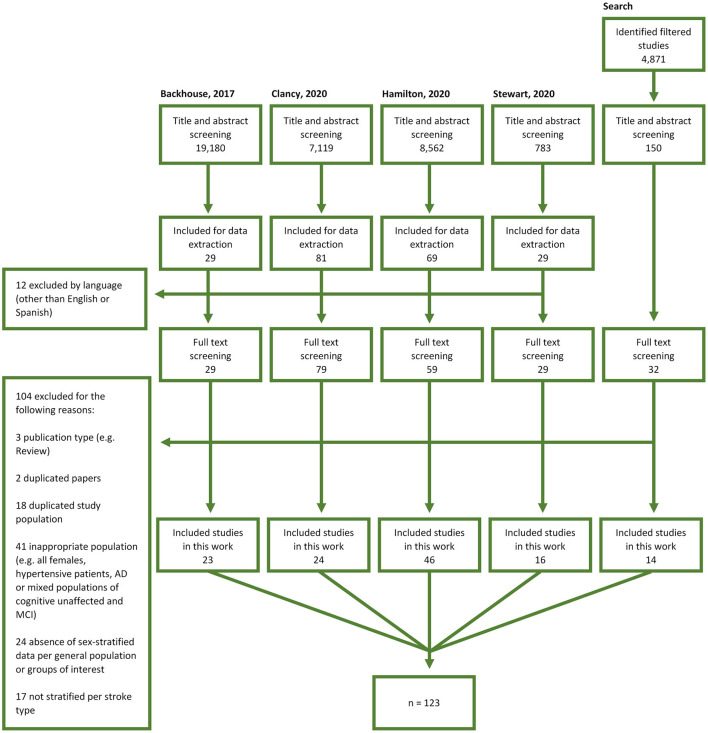
Study selection flow diagram. AD, Alzheimer's Disease; MCI, mild cognitive impairment.

We found 241 relevant journal articles in the four published systematic reviews and the independent search. After filtering by language, full texts of 228 publications were assessed against inclusion/exclusion criteria (Figure 1). We extracted data and meta-analyzed 123 studies that met the inclusion/exclusion criteria (*n* = 36,910 total participants) (24–146). Two studies included genetic SVD (CADASIL) (79, 83) and 121 studies included sporadic SVD (characteristics of the included studies are summarized in Supplementary Table 1). Studies were conducted from 1989 to 2020 in 23 countries across six continents (Europe 43; Asia 39; North America 35; South America 3; Australia 2; Africa 1).

None of the included studies reported data regarding non-binary participants. Hence, for simplicity and without prejudice, sex ratios are referred to as male:female ratios.

### Trends Across Study Settings

**Figure 2:**
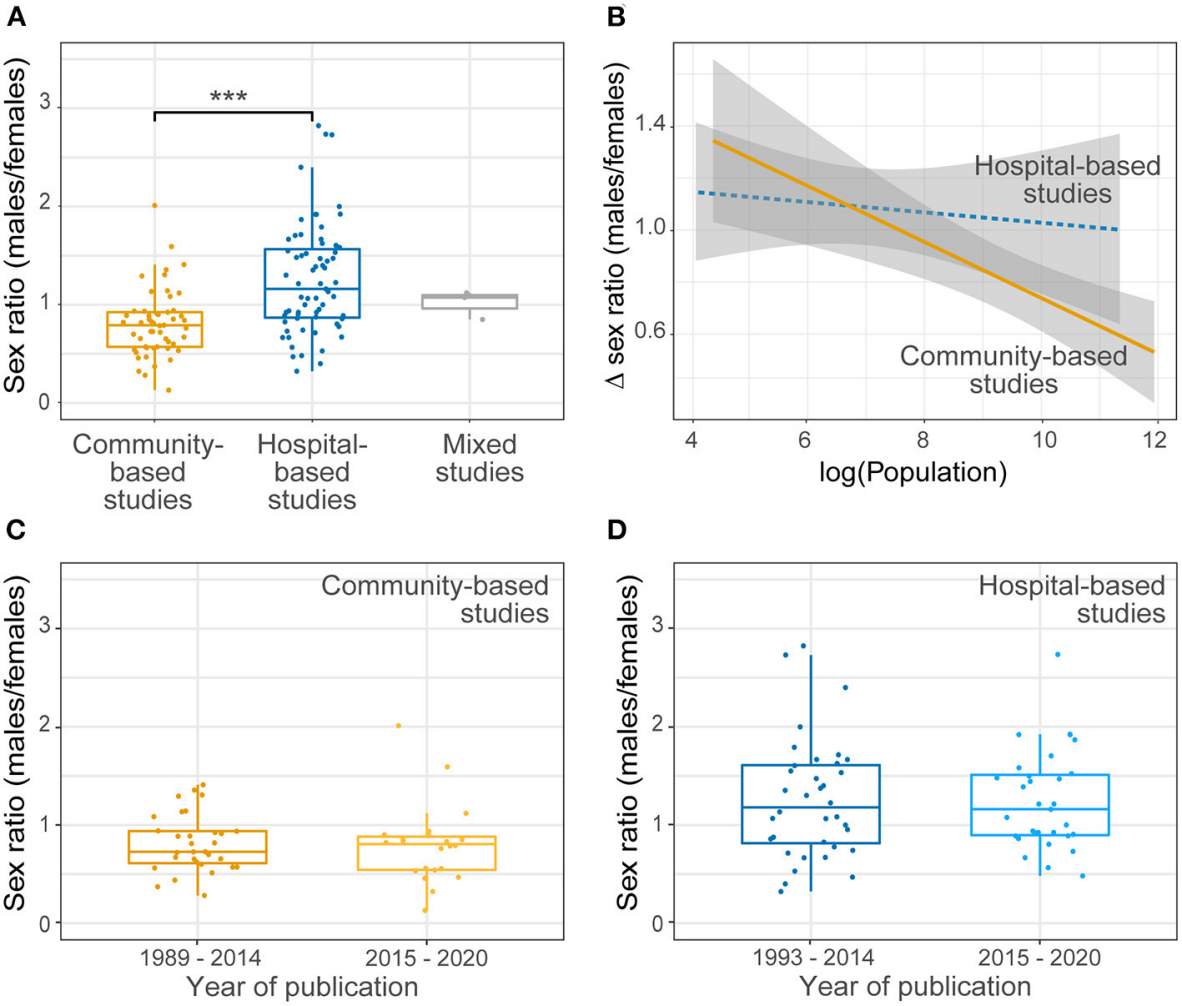
Sex ratio of SVD studies across study setting and time. **(A)** Comparison of sex ratios per study type. Significant differences were found between sex ratios of community-based (CB) and hospital-based (HB) studies (*p*_corrected_ < 0.001). **(B)** Correlation between the sex ratio difference and the size of the recruited sample. Δ sex ratio = |sex ratio of general population – sex ratio of each study|. Given that the mean age of the participants of the included studies was 67, general population sex ratio corresponds to 70-year old population (89 males per 100 females) (22). There was a negative correlation between Δ sex ratio and the size of the population recruited in community studies (yellow, rho_Spearman_ = −0.46, *p* < 0.001) but not in hospital studies (blue, rho_Spearman_ = −0.10, *p* = 0.43). **(C,D)** Comparison of sex ratios across time. No significant differences were found between sex ratios of recent studies compared with those previously published considering all included studies (*n*_2015−2020_ = 53 vs. *n*_1989−2014_ = 67, *U* = 1,814, *p* = 0.75), **(C)** CB studies (*n*_2015−2020_ = 22 vs. *n*_1989−2014_ = 31, *U* = 372, *p* = 0.58) or **(D)** HB studies (*n*_2015−2020_ = 31 vs. *n*_1993−2014_ = 36, *U* = 551, *p* = 0.93). ***p < 0.001.

Our literature search retrieved 53 community-based (*n* = 29,323 participants) (24–76), 67 hospital-based (*n* = 7,337 participants) (77–143), and three mixed studies (*n* = 250 participants) (144–146). The global sex ratio of all included studies was 0.92 (0.65). Sex ratios differed across study setting (H = 24.35, df = 2, *p* < 0.001), being greater in hospital-based studies (i.e., more males than females) than in community-based studies: 1.16 (0.70) vs. 0.79 (0.35), respectively (*p*_corrected_ < 0.001; Figure 2A). Considering that the mean age of the participants of the included studies was 67, the sex ratio of community-based studies was closer to the expected general population sex ratio (0.89 in a 70-year old population) (22) than that of the hospital-based studies.

Sex ratio varied with study size in community-based but not in hospital-based studies: in community-based studies, the sex ratio was closer to that of the general population when the sample size was larger (rho_Spearman_ = −0.46, *p* < 0.001; Figure 2B); there was no effect of sample size on sex ratio in hospital-based studies (rho_Spearman_ = −0.10; *p* = 0.43; Figure 2B).

### Trends Across Time

We classified studies per year of publication into recent (from 2015 to 2020) and previously published (until and including 2014) studies.

Considering all the included studies, there were no significant differences between sex ratios of recent studies compared with earlier publications (*U* = 1,814, *p* = 0.75). This finding was consistent after classifying by study type (*U* = 372, *p* = 0.58 in community-based studies; *U* = 551, *p* = 0.93 in hospital-based studies; Figures 2C,D). Mixed studies (144–146) were not included in this analysis since only three were retrieved by our literature search, all published recently.

### Trends Across Countries

**Figure 3:**
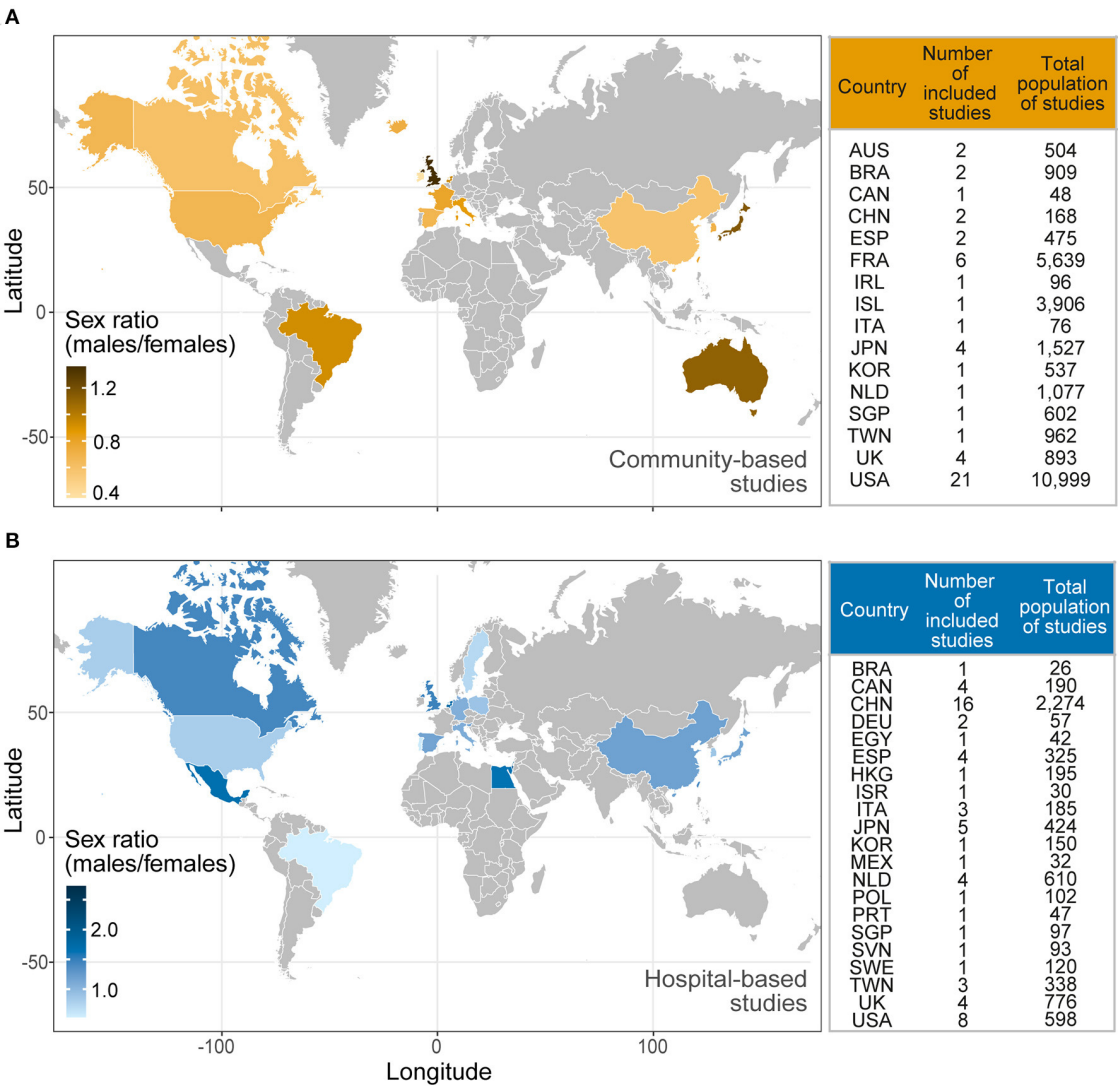
Sex ratio of SVD studies across the world. Colored world maps representing the mean sex ratio of the total number of participants of **(A)** community-based and **(B)** hospital-based studies. Darker shades in the color gradient correspond to higher sex ratios (i.e., more males than females). The tables on the right specify the country of recruited participants, the number of included studies and the total population of included studies per study type. Neither multicentre nor mixed studies were represented in these maps. AUS, Australia; BRA, Brazil; CAN, Canada; CHN, China; DEU, Germany; EGY, Egypt; ESP, Spain; FRA, France; HKG, Hong Kong; IRL, Ireland; ISR, Israel; ITA, Italy; JPN, Japan; KOR, Korea; MEX, Mexico; NLD, The Netherlands; POL, Poland; PRT, Portugal; SGP, Singapore; SVN, Slovenia; SWE, Sweden; TWN, Taiwan; UK, United Kingdom; USA, The United States of America.

We classified community-based and hospital-based studies by country of recruitment (Figure 3). For clarity, studies that recruited participants from more than one country (37, 63, 83, 109, 127) and mixed studies (144–146) were excluded.

Amongst community-based studies, the highest sex ratio was found in participants recruited from the United Kingdom [1.36 (0.19), four studies, *n* = 893] and the lowest in participants recruited from the Republic of Ireland (0.37, one study, *n* = 96; Figure 3A). The largest recruited population came from the USA (21 studies, *n* = 10,999 participants), with a median sex ratio of 0.67 (0.36). Regarding hospital-based studies, the highest sex ratio was found in participants recruited from Singapore (2.73, one study, *n* = 97) and the lowest in participants recruited in Brazil (0.53, one study, *n* = 26; Figure 3B). The largest recruited population came from China (16 studies, *n* = 2,274 participants), with a median sex ratio of 1.08 (0.48).

There were no obvious regional trends across countries for sex ratio vs. the total number of participants for either community-based or hospital-based studies.

### Severity and Presentation of SVD

**Figure 4:**
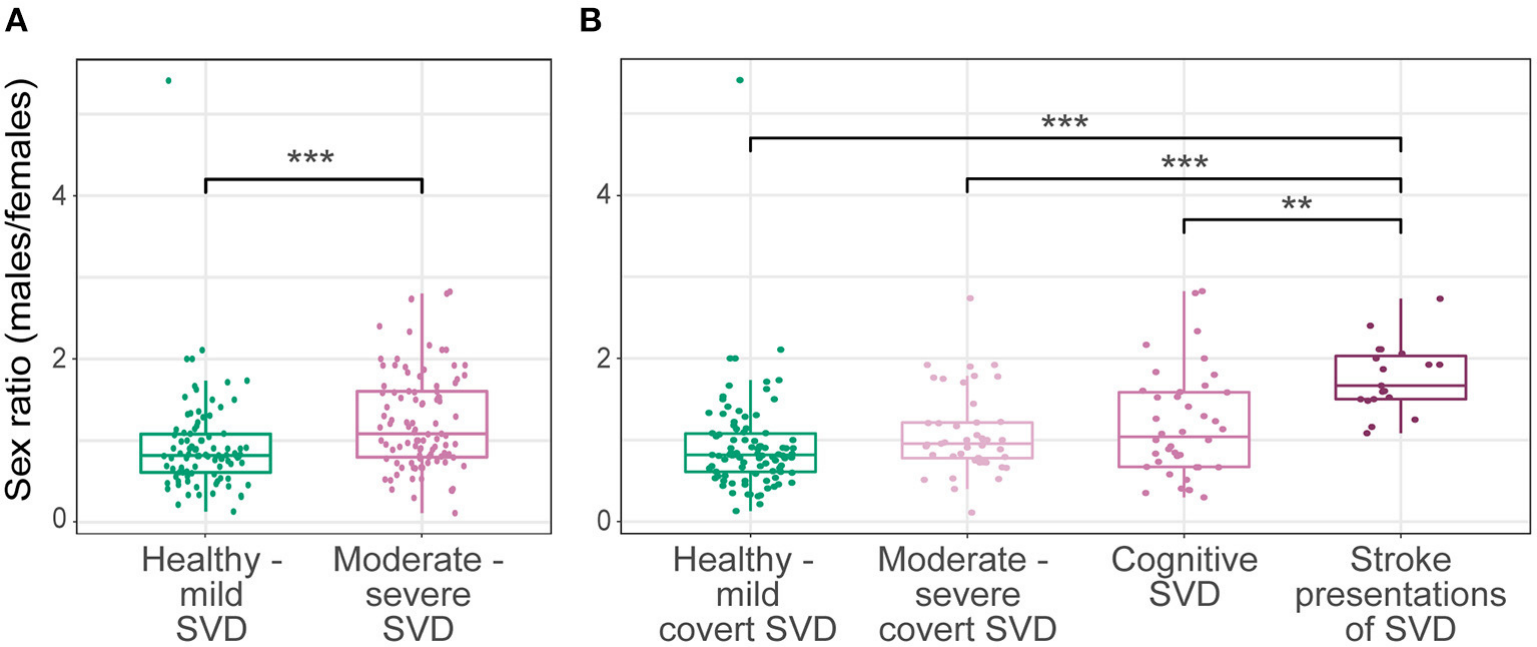
Sex ratio across SVD severity and presentation. Sex ratio of healthy to mild SVD compared with **(A)** moderate to severe SVD and **(B)** stratified moderate to severe SVD. Significant differences were found between SVD severity groups i.e., sex ratios of healthy to mild SVD and moderate to severe SVD (A; *U* = 3,031, *p* < 0.001). Significant differences were also found between SVD presentation groups (H = 36.58, df = 3, *p* < 0.001) i.e., stroke presentations of SVD compared with healthy to mild covert SVD, moderate to severe covert SVD or cognitive SVD (B; *p*_corrected_ < 0.001, *p*_corrected_ < 0.001, *p*_corrected_ = 0.003, respectively). **p < 0.01, ***p < 0.001.

The included studies enrolled *n* = 25,972 healthy to mild SVD participants (no SVD presentation or mild covert SVD, Table 2) and *n* = 10,938 moderate to severe SVD participants (clinical presentation and/or high radiological burden of SVD, Table 2). The sex ratio was higher in healthy to mild SVD [1.08 (0.81)] than in moderate to severe SVD [0.82 (0.47)], *U* = 3,031, *p* < 0.001 (Figure 4A).

We further classified moderate to severe SVD participants into cognitive or stroke presentations or moderate to severe covert SVD (Table 2, Figure 4B). We excluded CADASIL studies due to insufficient data (79, 83). Sex ratios differed significantly between SVD presentations (H = 36.58, df = 3, *p* < 0.001). Participants with stroke showed the highest sex ratio, 1.67 (0.53), compared with healthy to mild covert SVD [0.82 (0.47), *p*_corrected_ < 0.001], cognitive SVD [1.03 (0.91), *p*_corrected_ = 0.003], and moderate to severe covert SVD [0.96 (0.44), *p*_corrected_ < 0.001].

Since community-based studies recruited mostly healthy participants and presented lower sex ratios, we repeated the severity analysis restricted to hospital-based studies (77–143), and found similar trends. Across SVD severity, the sex ratio was higher in moderate to severe SVD [1.26 (0.87)] than in healthy to mild SVD [0.90 (0.58)], *U* = 1,240, *p* < 0.001. Across SVD presentations, the sex ratio was higher in stroke presentations [1.67 (0.55)] than in healthy to mild covert SVD [0.90 (0.58), *p*_corrected_ < 0.001], cognitive SVD [1.11 (0.81), *p*_corrected_ = 0.037], and moderate to severe covert SVD [1.13 (0.87) *p*_corrected_ = 0.02], H = 21.82, df = 3, *p* < 0.001.

### Age and Risk Factors for SVD

Only 10 studies (38, 64, 65, 69, 73, 81, 102, 111, 136, 143) (2,953 participants) provided sufficient data to calculate the sex-stratified participants' age. There was no significant difference in median age between total recruited males [63.78 (9.71)] and females [64.45 (13.71), *U* = 49.5, *p* > 0.99].

Only two studies (73, 81) provided data to calculate sex-stratified SVD risk factors (hypertension and smoking), which were insufficient to perform further analyses.

### Quality Assessment

The median study quality score was 5.5 (1). As a sensitivity analysis, quantitative analyses were re-run excluding all studies with a quality score <5.5/8. All the trends observed in the total included studies were consistent in the subset of higher-quality studies (score ≥ 5.5/8; Supplementary Table 2).

## Discussion

This meta-analysis of 123 studies (24–146) including 36,910 participants demonstrates sex differences in SVD across study settings, by SVD severity and presentation. A greater male-to-female ratio was found in hospital-based (77–143) compared to community-based studies (24–76) (Figure 2), and in moderate to severe SVD, particularly in stroke presentations (Figure 4). The pattern was consistent across recent (2015–2020) and previous (1989–2014) studies (Figures 2C,D), and world regions (Figure 3). To the best of our knowledge, this is the first systematic review and meta-analysis to explore sex differences in SVD and has important implications. The apparent presence of more severe SVD in males, particularly amongst those presenting with stroke, may indicate differences in risk factor exposures, susceptibility to SVD, adherence to risk factor interventions, or differences in study recruitment. Awareness of these differences, which were robust to study location and population, may help inform approaches to mitigate the long-term effects of covert SVD and for secondary prevention of SVD-related stroke. Unfortunately, very few studies reported risk factor differences between males and females and even age was not commonly reported. Furthermore, future studies should report male and female demographics, risk factors and outcomes, not just total sample data.

The different sex ratios between community-based and hospital-based studies may reflect differences in recruitment in these settings. Typically, females are older and have more disability at stroke onset (7), which may affect study eligibility. For example, ischemic stroke patients older than 80 years have higher rates of disability following thrombolysis treatment (147) and are less likely to be recruited into stroke trials (148). Furthermore, women with stroke may present with non-traditional symptoms like altered mental status (149), which could be overlooked or misdiagnosed (150, 151), and are important since atypical and neuropsychiatric symptoms are increasingly recognized to associate with SVD (15). Sex differences in clinical presentations are also present in dementia (152) but none of the included studies reported these in VaCI or VaD. Moreover, informal carers of dependent persons in the UK are more likely to be middle-aged women with multiple roles until later life (70+) (153). Thus, females may be reluctant to participate in studies due to care responsibilities or may normalize their early symptoms while providing care. Caregiving roles vary by country (154), socioeconomic status and culture of care (155), which might explain why more females seemed to participate in Chinese hospital-based studies compared with the UK or Canada (Figure 3B) since Chinese males are traditionally the predominant caregivers for older parents (156). Some of the aforementioned factors that may alter female recruitment to SVD studies have recently been highlighted as contributors to lower enrolment of women in stroke clinical trials more generally (157).

It could also be that SVD is more prevalent and/or severe in males than in females, increasing the likelihood of males becoming participants in studies investigating severe SVD. In support of this, male-sex was an independent predictor of severity of SVD in an adjusted analysis, albeit in a 62% male population (20). Similarly, a greater prevalence of stroke, higher cognitive impairment and cerebral atrophy have been reported in men with CADASIL (158). Sex differences can be driven by sex-specific biological factors e.g., sexual dimorphism in endothelial function (159). In premenopausal females, oestrogens enhance endothelial production of vasodilator factors (160). This may explain young males having greater vasoconstrictor tone compared to pre-menopausal females (159) and male endothelial function becoming suboptimal under certain insults. We found no differences in age between recruited males and females, although fewer data were available for this analysis. Different lifestyle-related risk factors could also contribute to the sex-specific severity of SVD, e.g., utilization of preventative health care services, smoking, or hypertension. Interestingly, the prevalence of smoking and hypertension is higher among males in most countries (161, 162), varying with ethnicity (163). Unfortunately, there were insufficient data to determine if sex-specific risk factor effects were driving the sex ratio difference in SVD severity and presentation.

The unequal sex ratios found here may be explained by factors with different contributions across different settings, evidenced by the different effect of study size on sex ratio within community-based and hospital-based studies (Figure 2B), or in the context of higher SVD severity and stroke presentations. The lack of difference between sex ratios of recent and earlier studies (Figures 2C,D) suggests that the same factors may have operated long term.

The implications for future research and clinical practice are varied and important. The lack of sex-stratified data, previously reported in brain structural studies (164) and aging research (10), hampers translational research and personalized care. Results should be reported and analyzed by sex, especially when biological factors, treatments or social disparities may differ between sexes (11). This was addressed recently (165, 166) in support of the Sex and Gender Equity in Research (SAGER) guidelines (167) and the European Commission second report on Gendered Innovations (168), which provides guidance for researchers to incorporate sex, gender and intersectional analysis across several research topics. Future studies should also identify and try to avoid recruitment bias, explore whether SVD is more frequently underestimated or misdiagnosed in females and investigate reasons why males may be more severely affected. Larger sample sizes may help to reduce sampling variability at least within community-based studies with a majority of functionally healthy individuals (Figure 2B). If the disease in females is going unrecognized, doctors and the public could be educated to better recognize atypical symptoms in females. If males are more severely affected or exposed to certain lifestyle factors, trials may need to target drivers of males' vulnerability and health promotion campaigns could be designed to have more impact on males.

This study had limitations. First, it was not possible to explore common risk factors of SVD due to the scarcity of sex-stratified data. Also, other risk factors and their differences between sexes were not explored (e.g., lower educational attainment, associated with increased risk of SVD in later life) (13). Most studies are from industrialized countries (Figure 3), so our results might not fully represent other populations. Since this study relied on studies' own criteria for SVD severity, future explorations could investigate heterogeneity in study criteria and attempt further standardization efforts. Future work could also explore the sex-stratified functional status of participants, which may affect eligibility criteria and result in exclusion of females who are more functionally disabled (6, 8, 169).

The study also has strengths. We included studies from four recent systematic reviews, which covered different aspects of SVD (and therefore likely different literatures), each had assessed a large literature, and the source files were immediately available to us. We did not use our older systematic reviews as the included literature would not have been so up to date or comprehensive. We hope that our approach might encourage other groups to evaluate their high-quality systematic reviews in the same way. A broad approach was taken to capture changes across time, study settings, different cultural or ethnic groups, SVD severity and presentation. The included studies were conducted from 1989 to 2020, recruited 36,910 participants from the community and/or hospitals in 23 countries across six continents, and explored a wide range of SVD radiological features and presentations. Our results highlight sex-specific variability in study participation, SVD severity, and presentation. These findings are relevant for future research and clinical practice, but much more work is needed to unmask sex-specific biological and social disparities and disentangle their contributions to sex differences in SVD.

## Data Availability Statement

The original contributions presented in the study are included in the article/Supplementary Material, further inquiries can be directed to the corresponding author/s.

## Author Contributions

LJ-S carried out the independent literature search, extracted the data, performed the meta-analyses, and drafted the manuscript. OKLH, EVB, UC, and CRS carried out the literature search of their corresponding systematic reviews and provided their databases and reviewed and edited the manuscript. MSS co-supervised one of the systematic reviews (conducted by CRS) and checked and edited the manuscript. FND co-supervised one of the systematic reviews (conducted by UC) and checked and edited the manuscript. JMW conceived and managed the project, designed the protocol, checked the search strategy, supervised the contributing meta-analyses, reviewed uncertain articles, advised on the meta-analysis and interpretation of data, and reviewed and edited the manuscript. The final draft of the manuscript was approved by all authors.

## Funding

This research was funded, in part, by the Wellcome Trust [Grant No. 108890/Z/15/Z]. For the purpose of open access, the author has applied a CC BY public copyright license to any Author Accepted manuscript version arising from this submission. LJ-S is a Translational Neuroscience PhD student funded by Wellcome (108890/Z/15/Z). OKLH was a Translational Neuroscience PhD student funded by the College of Medicine and Veterinary Medicine at the University of Edinburgh. OKLH was supported by a Translational Neuroscience PhD student funded by the College of Medicine and Veterinary Medicine at the University of Edinburgh. UC was funded by a Chief Scientist Office of Scotland Clinical Academic Fellowship (CAF/18/08) and Stroke Association Princess Margaret Research Development Fellowship (2018). EVB was funded by the Sackler Foundation, the Stroke Association, British Heart Foundation and Alzheimer's Society through the R4VaD Study. MSS was funded by the Fondation Leducq (ref no. 16 CVD 05) and EU Horizon 2020 (PHC-03-15, project No 666881, SVDs^@^Target) and the MRC UK Dementia Research Institute at the University of Edinburgh (UK DRI LTD, funded by the UK Medical Research Council, Alzheimer's Society and Alzheimer's Research UK). FND was funded by a Stroke Association Garfield Weston Foundation (TSALECT 2015/04) Senior Clinical Lectureship and NHS Research Scotland. JMW was funded by the Stroke Association, British Hearth Foundation, Row Fogo Charitable Trust, Fondation Leducq (Perivascular Spaces Transatlantic Network of Excellence), and EU Horizon 2020 (SVDs^@^Target) and the MRC UK Dementia Research Institute at the University of Edinburgh (UK DRI LTD, funded by the UK Medical Research Council, Alzheimer's Society and Alzheimer's Research UK). All authors hold grants from government/charitable agencies. The funding sources had no role in the study design, execution, analysis, interpretation of the data, decision to publish, or preparation of the manuscript.

## Conflict of Interest

The authors declare that the research was conducted in the absence of any commercial or financial relationships that could be construed as a potential conflict of interest. The reviewer CC declared a past co-authorship with one of the authors JMW to the handling editor.

## Publisher's Note

All claims expressed in this article are solely those of the authors and do not necessarily represent those of their affiliated organizations, or those of the publisher, the editors and the reviewers. Any product that may be evaluated in this article, or claim that may be made by its manufacturer, is not guaranteed or endorsed by the publisher.

## Abbreviations

CADASIL: cerebral autosomal dominant arteriopathy with subcortical infarcts and leukoencephalopathy
CMBs: cerebral microbleeds
MRI: magnetic resonance imaging
ICH: intracerebral hemorrhage
SVD: cerebral small vessel disease
VaD: vascular dementia
VaCI: vascular cognitive impairment
WMH: white matter hyperintensities.
