# Supplementary material for: Sex Differences in Cerebral Small Vessel Disease: A Systematic Review and Meta-Analysis
Source: Front Neurol. 2021 Oct 28;12:756887. doi: 10.3389/fneur.2021.756887 (PMC8581736; doi:10.3389/fneur.2021.756887)
Supplement: Supplementary file 1 [file Data_Sheet_1.docx]

**Supplementary Material**

**Supplementary Table 1. Characteristics of all included studies.**

**Supplementary Table 2. Results of quantitative tests excluding studies with quality scores < 5.5/8.**

**References.**

**Table 1. Characteristics of all included studies.**

| **Study (Primary author, year)** | **Community-based (C), hospital-based (H) or mixed (M)** | **Country of recruited participants** | **SVD features of selected participants (original definitions used)** | **Number of total participants** | **Mean age of total participants** | **Males** | | | | **Females** | | | |
| --- | --- | --- | --- | --- | --- | --- | --- | --- | --- | --- | --- | --- | --- |
|  |  |  |  |  |  | **n** | **Mean age** | **% Hypertension** | **% Ever smoking** | **n** | **Mean age** | **% Hypertension** | **% Ever smoking** |
| Kim, 2020 (1) | C | USA | Healthy participants  MCI | 38 | 68.31 | 7  11 | NA | NA | NA | 14  6 | NA | NA | NA |
| Cedres, 2019 (2) | C | Spain | Healthy participants | 416 | 58.5 | 190 | NA | NA | NA | 226 | NA | NA | NA |
| Dolui, 2019 (3) | C | USA | Healthy participants | 497 | 53.21 | 224 | NA | NA | NA | 273 | NA | NA | NA |
| Legdeur, 2019 (4) | C | USA | Healthy participants | 141 | 94.3 | 45 | NA | NA | NA | 96 | NA | NA | NA |
| Mishra, 2019 (5) | C | France | Minimal SVD  Extensive SVD | 580 | 64.75 | 98  109 | NA | NA | NA | 223  150 | NA | NA | NA |
| Puzo, 2019 (6) | C | USA | Healthy participants | 465 | 68.9 | 146 | NA | NA | NA | 319 | NA | NA | NA |
| Staffaroni, 2019 (7) | C | USA | Healthy participants | 161 | 69.9 | 73 | NA | NA | NA | 88 | NA | NA | NA |
| Tsapanou, 2019 (8) | C | USA | Healthy participants | 562 | 54 | 243 | NA | NA | NA | 319 | NA | NA | NA |
| Yao, 2019 (9) | C | Japan | Healthy participants | 259 | 68.4 | 122 | NA | NA | NA | 137 | NA | NA | NA |
| Croall, 2018 (10) | C | UK | Severe SVD (with confirmed LS) | 70 | 69.45 | 43 | NA | NA | NA | 27 | NA | NA | NA |
| Kuriyama, 2018 (11) | C | Japan | Controls  Deep WMLs Faz 1  Deep WMLs Faz 2  Deep WMLs Faz 3 | 280 | 70.8 | 45  92  40  10 | NA | NA | NA | 23  42  22  6 | NA | NA | NA |
| Puglisi, 2018 (12) | C | Italy | WMLs Faz 1  WMLs Faz 2  WMLs Faz 3 | 76 | 72.5 | 7  15  13 | NA | NA | NA | 13  17  11 | NA | NA | NA |
| Shokouhi, 2018 (13) | C | USA | Controls  MCI | 265 | 64.0 | 19  74 | NA | NA | NA | 61  111 | NA | NA | NA |
| Van Rooden, 2018 (14) | C | Netherlands and USA | Controls  Subjective cognitive decline | 67 | 68.0 | 17  7 | NA | NA | NA | 25  18 | NA | NA | NA |

| Bahrani, 2017 (15) | C | USA | Healthy participants | 26 | 77.8 | 3 | 77.0 | NA | NA | 23 | 77.8 | NA | NA |
| --- | --- | --- | --- | --- | --- | --- | --- | --- | --- | --- | --- | --- | --- |
| Squarzoni, 2017 (16) | C | Brazil | Controls  Silent vascular brain lesions | 234 | 73.91 | 88  25 | NA | NA | NA | 89  32 | NA | NA | NA |
| Shi, 2017 (17) | C | China | Healthy participants. | 69 | 70.78 | 24 | NA | NA | NA | 45 | NA | NA | NA |
| Xu, 2017 (18) | C | Singapore | Controls  1 CMB  Multiple CMB | 802 | 70.3 | 233  67  69 | NA | NA | NA | 289  95  49 | NA | NA | NA |
| Chung, 2016 (19) | C | Taiwan | Healthy participants | 962 | 62.5 | 425 | NA | NA | NA | 537 | NA | NA | NA |
| Promjunyakul, 2016 (20) | C | USA | Healthy participants | 82 | 84 | 20 | NA | NA | NA | 62 | NA | NA | NA |
| Vemuri, 2015 (21) | C | USA | Controls  Vascular pathology | 267 | 76.67 | 93  48 | NA | NA | NA | 85  41 | NA | NA | NA |
| Yamawaki, 2015 (22) | C | Japan | Mild DWMH Faz 0-1  Moderate DWMH Faz 2  Severe DWMH Faz 3 | 688 | 76.5 | 198  68  37 | NA | NA | NA | 240  108  37 | NA | NA | NA |
| Annweiler, 2014 (23) | C | France | Subjective memory complaint | 133 | 71.6 | 75 | NA | NA | NA | 58 | NA | NA | NA |
| Mortamais, 2014 (24) | C | France | Low WMLs < 0.3 ml  Mild WMLs 0.3-1.5 ml  Severe WMLs > 1.5 ml | 500 | 71 | 77  86  72 | NA | NA | NA | 95  85  85 | NA | NA | NA |
| Sarabia-Cobo, 2014 (25) | C | Spain | MCI | 59 | 77.80 | 20 | NA | NA | NA | 39 | NA | NA | NA |
| Sims, 2014 (26) | C | USA | Healthy participants | 172 | 64.43 | 99 | NA | NA | NA | 73 | NA | NA | NA |
| Sun, 2014 (27) | C | China | Controls  Mild WMLs | 99 | 64.11 | 16  22 | NA | NA | NA | 33  28 | NA | NA | NA |
| Wiegman, 2014 (28) | C | USA | Controls  CMBs | 243 | 84.50 | 51  23 | NA | NA | NA | 125  44 | NA | NA | NA |
| Farfel, 2013 (29) | C | Brazil | Healthy participants | 675 | 74.0 | 322 | NA | NA | NA | 353 | NA | NA | NA |

| Minn, 2013 (30) | C | South Korea | Controls  WM changes or SI | 537 | 63 | 122  99 | NA | NA | NA | 219  97 | NA | NA | NA |
| --- | --- | --- | --- | --- | --- | --- | --- | --- | --- | --- | --- | --- | --- |
| Nebes, 2013 (31) | C | USA | Controls  WMH | 66 | NA | 15  9 | NA | NA | NA | 25  17 | NA | NA | NA |
| Valdés-Hernández, 2013 (32) | C | UK | Healthy participants | 634 | 72.7 | 337 | NA | NA | NA | 297 | NA | NA | NA |
| Bartley, 2012 (33) | C | Ireland | Controls  Subjective memory complaints | 96 | 62.5 | 11  15 | NA | NA | NA | 33  37 | NA | NA | NA |
| Salarirad, 2011 (34) | C | UK | Healthy participants | 106 | 78.3 | 62 | NA | NA | NA | 44 | NA | NA | NA |
| Stewart, 2011 (35) | C | France | Healthy participants | 1,793 | 72.4 | 710 | NA | NA | NA | 1,083 | NA | NA | NA |
| Villeneuve, 2011 (36) | C | Canada | Controls  MCI with confluent WMLs | 48 | 72.05 | 7  11 | NA | NA | NA | 20  10 | NA | NA | NA |
| Qiu, 2010 (37) | C | Iceland | Controls  CMBs | 3,906 | 76 | 1,399  246 | NA | NA | NA | 2,057  204 | NA | NA | NA |
| Godin, 2009 (38) | C | France | Healthy participants | 1,792 | 72.4 | 708 | NA | NA | NA | 1,084 | NA | NA | NA |
| Anderson, 2008 (39) | C | Australia | Controls  First-ever lacunar syndrome | 60 | 68.63 | 16  16 | NA | NA | NA | 14  14 | NA | NA | NA |
| Miranda, 2008 (40) | C | Multicenter | LA without memory impairment  LA with memory impairment | 638 | 74.1 | 107  180 | NA | NA | NA | 132  219 | NA | NA | NA |
| Christensen, 2007 (41) | C | Australia | Healthy participants | 444 | 62.64 | 231 | 62.65 | NA | NA | 213 | 62.63 | NA | NA |
| Schretlen, 2007 (42) | C | USA | Healthy participants | 177 | 35.97 | 85 | 62.1 | NA | NA | 92 | 58.8 | NA | NA |
| Au, 2006 (43) | C | USA | Non-large WMH  Large WMH | 1,819 | 61.15 | 745  109 | NA | NA | NA | 834  131 | NA | NA | NA |
| Elkins, 2006 (44) | C | USA | Healthy participants | 3,622 | 75.06 | 1,524 | NA | NA | NA | 2,098 | NA | NA | NA |
| Wright, 2005 (45) | C | USA | Healthy participants | 259 | 64.8 | 116 | NA | NA | NA | 143 | NA | NA | NA |
| Deary, 2003 (46) | C | UK | Healthy participants | 83 | 78 | 47 | 78 | NA | NA | 36 | 78 | NA | NA |
| Dufouil, 2003 (47) | C | France | No lesion or mild WMH  Moderate WMH  Severe WMH | 841 | 69 | 145  149  58 | NA | NA | NA | 175  229  85 | NA | NA | NA |
| Tsukishima, 2001 (48) | C | Japan | Controls  With WML  With SI  Both WML and SI | 300 | 72.9 | 112  7  15  11 | NA | NA | NA | 121  8  20  6 | NA | NA | NA |
| De Groot, 2000 (49) | C | Netherlands | Controls  WMLs | 1,077 | 72.21 | 22  500 | NA | NA | NA | 32  523 | NA | NA | NA |
| Liao, 1997 (50) | C | USA | Controls  Mild WMLs  Moderate WMLs  Severe WMLs | 1,921 | 62 | 97  372  196  106 | Black males: 62  White males: 63 | Black males: 56  White males: 38 | Black males: 69  White males: 76 | 183  586  248  133 | Black females: 61  White females: 63 | Black females: 68  White females: 31 | Black females: 69  White females: 46 |
| Boone, 1992 (51) | C | USA | Healthy participants | 100 | 62.8 | 36 | NA | NA | NA | 64 | NA | NA | NA |
| Tupler, 1992 (52) | C | USA | Controls  DWMH | 66 | 61.8 | 15  9 | NA | NA | NA | 33  9 | NA | NA | NA |
| Rao, 1989 (53) | C | USA | Controls  LA | 50 | 43.7 | 10  1 | NA | NA | NA | 30  9 | NA | NA | NA |
| Jin, 2020 (54) | H | China | SIVD | 73 | 48.0 | 48 | NA | NA | NA | 25 | NA | NA | NA |
| Zhou, 2020 (55) | H | China | LI grade 0  LI grade 1  LI grade 2  LI grade 3 | 175 | 76.36 | 22  24  26  22 | NA | NA | NA | 23  20  21  17 | NA | NA | NA |
| Jokumsen-Cabral, 2019 (56) | H | Portugal | Controls  CADASIL | 47 | 57.85 | 6  11 | NA | NA | NA | 14  16 | NA | NA | NA |
| Kate, 2019 (57) | H | Canada | ICH | 71 | 69 | 52 | NA | NA | NA | 19 | NA | NA | NA |
| Liang, 2019a (58) | H | Hong Kong | First-ever IS with lacunae  First-ever IS with CMBs | 195 | 66.17 | 73  54 | 64.9 | 59.5 | 59.1 | 44  24 | 68.0 | 72.6 | 7.5 |
| Liang, 2019b (59) | H | China | Controls  LS | 831 | NA | 238  245 | NA | NA | NA | 187  161 | NA | NA | NA |
| Ling, 2019 (60) | H | France and Germany | CADASIL | 160 | 41.0 | 76 | NA | NA | NA | 84 | NA | NA | NA |
| Liu, 2019a (61) | H | China | Controls  SIVD without CI  SIVD with CI | 81 | 69.54 | 10  16  16 | NA | NA | NA | 17  9  13 | NA | NA | NA |
| Liu 2019b (62) | H | China | Subcortical infarcts | 50 | 52.6 | 30 | NA | NA | NA | 20 | NA | NA | NA |
| Liu, 2019c (63) | H | China | Controls  SVD without CI  SVD with CI | 66 | 64.36 | 13  10  10 | NA | NA | NA | 12  11  10 | NA | NA | NA |
| Manso-Calderón, 2019 (64) | H | Spain | SVaD | 184 | 80.3 | 82 | NA | NA | NA | 102 | NA | NA | NA |
| Oudeman, 2019 (65) | H | Netherlands | VaCI | 58 | 68.9 | 35 | NA | NA | NA | 23 | NA | NA | NA |
| Reginold, 2019 (66) | H | Canada | WMH | 31 | 70.5 | 17 | NA | NA | NA | 14 | NA | NA | NA |
| Rudilosso, 2019 (67) | H | Spain | Subcortical infarcts | 67 | 66.3 | 40 | NA | NA | NA | 27 | NA | NA | NA |
| Staszewski, 2019 (68) | H | Poland | LS  VaD | 102 | 63.88 | 35  13 | NA | NA | NA | 17  37 | NA | NA | NA |
| Tsai, 2019 (69) | H | Taiwan | ICH | 257 | 63.15 | 162 | NA | NA | NA | 95 | NA | NA | NA |
| Wu, 2019 (70) | H | China | SIVD | 73 | 65.71 | 48 | NA | NA | NA | 25 | NA | NA | NA |
| Yu, 2019 (71) | H | Canada | Controls  SIVD | 54 | 71.7 | 11  14 | NA | NA | NA | 14  15 | NA | NA | NA |
| Zhang, 2019 (72) | H | China | Controls  Amnesic MCI with high grade WMH | 186 | 67.61 | 46  44 | NA | NA | NA | 44  52 | NA | NA | NA |
| Ishibashi, 2018 (73) | H | Japan | MCI without WMH  MCI with WMH | 75 | 78.1 | 11  25 | NA | NA | NA | 18  21 | NA | NA | NA |
| Kim, 2018 (74) | H | USA | Controls  Subcortical VaCI | 80 | 77.73 | 9  17 | NA | NA | NA | 10  44 | NA | NA | NA |
| Lisiecka-Ford, 2018 (75) | H | UK | SVD corresponding LS and Faz ≥ 2 | 114 | 70 | 75 | NA | NA | NA | 39 | NA | NA | NA |
| Anor, 2017 (76) | H | Canada | VaD | 34 | 75.3 | 16 | NA | NA | NA | 18 | NA | NA | NA |
| Yuan, 2017 (77) | H | China | Controls  LA | 100 | 70.45 | 19  21 | NA | NA | NA | 31  29 | NA | NA | NA |
| Zhong, 2017 (78) | H | China | WMH | 75 | 67.05 | 36 | NA | NA | NA | 39 | NA | NA | NA |
| Bella, 2016 (79) | H | Italy | Controls  VaCI | 45 | 66.21 | 9  10 | 66.05 | NA | NA | 11  15 | 66.27 | NA | NA |
| Hashimoto, 2016 (80) | H | Japan | CMBs median < 5  CMBs median > 5 | 22 | 69 | 5  8 | NA | NA | NA | 5  4 | NA | NA | NA |
| Hsu, 2016 (81) | H | Taiwan | Controls  VaMCI | 50 | 66.32 | 10  14 | NA | NA | NA | 20  6 | NA | NA | NA |
| Turk, 2016 (82) | H | Slovenia | Controls  Ischemic LA | 93 | 53.55 | 22  29 | NA | NA | NA | 18  24 | NA | NA | NA |
| Brookes, 2015 (83) | H | UK | Controls  Lacunar syndrome | 499 | 62.89 | 164  133 | NA | NA | NA | 139  63 | NA | NA | NA |
| Hsu, 2015 (84) | H | Taiwan | MCI | 31 | 75.4 | 19 | NA | NA | NA | 12 | NA | NA | NA |
| Brookes, 2014 (85) | H | UK | Controls  Lacunar syndrome plus LI | 125 | 68.68 | 36  25 | NA | NA | NA | 44  20 | NA | NA | NA |
| Delrieu, 2014 (86) | H | Multicenter | MCI | 65 | 74.8 | 48 | NA | NA | NA | 17 | NA | NA | NA |
| Ledesma-Amaya, 2014 (87) | H | Mexico | Controls  LI | 32 | 63.88 | 10  10 | NA | NA | NA | 6  6 | NA | NA | NA |
| Pinkhardt, 2014 (88) | H | Germany | DWMH Faz 1 ^a^  DWMH Faz 2  DWMH Faz 3 | 25 | 74.68 | 2  3  3 | 73.88 | NA | NA | 2  9  6 | 75.06 | NA | NA |
| Zi, 2014 (89) | H | China | Controls  PWMH | 32 | 61.73 | 7  7 | NA | NA | NA | 9  9 | NA | NA | NA |
| Deguchi, 2013 (90) | H | Japan | Controls  LI | 181 | 72.18 | 60  50 | NA | NA | NA | 45  26 | NA | NA | NA |
| Fang, 2013 (91) | H | China | Controls  SI  CMB  Both SI and CMB | 227 | 71.30 | 47  20  24  26 | NA | NA | NA | 44  26  17  23 | NA | NA | NA |
| Kim, 2013 (92) | H | USA | Subcortical VaMCI  Subcortical VaD | 127 | 73.8 | 23  28 | NA | NA | NA | 36  40 | NA | NA | NA |
| Narasimhalu, 2013 (93) | H | Singapore | LS without subjective CI  LS with subjective CI | 97 | 53 | 21  50 | NA | NA | NA | 9  17 | NA | NA | NA |
| Sudo, 2013 (94) | H | Brazil | Controls  VaMCI | 26 | 73.11 | 3  6 | NA | NA | NA | 8  9 | NA | NA | NA |
| Van Norden, 2013 (95) | H | Netherlands | SVD without subjective CI  SVD with subjective CI | 497 | 65.6 | 30  251 | NA | NA | NA | 17  199 | NA | NA | NA |
| Li, 2012 (96) | H | China | Controls  Ischemic LA | 40 | 65.45 | 12  11 | NA | NA | NA | 8  9 | NA | NA | NA |
| Quinque, 2012 (97) | H | Germany | Controls  Early cerebral microangiopathy | 32 | 64.42 | 13  7 | NA | NA | NA | 8  4 | NA | NA | NA |
| Yi, 2012 (98) | H | China | Controls  Subcortical VaMCI | 54 | 67.21 | 12  11 | NA | NA | NA | 16  15 | NA | NA | NA |
| Fernández, 2011 (99) | H | Spain | Controls  Subcortical VaMCI | 38 | 71.5 | 9  13 | NA | NA | NA | 10  6 | NA | NA | NA |
| Xiong, 2011 (100) | H | China | LS without cognitive complaints  LS with cognitive complaints | 75 | 70.75 | 20  19 | NA | NA | NA | 23  13 | NA | NA | NA |
| Hassan, 2010 (101) | H | Egypt | Controls  LI | 42 | 58.59 | 8  18 | NA | NA | NA | 4  12 | NA | NA | NA |
| Pascual, 2010 (102) | H | Spain | Controls  VWMD without dementia  VWMD with dementia | 36 | 80.1 | 6  6  6 | NA | NA | NA | 6  6  6 | NA | NA | NA |
| Seo, 2010 (103) | H | South Korea | Controls  Subcortical VaMCI  Subcortical VaD | 150 | 69.22 | 42  19  9 | NA | NA | NA | 54  15  11 | NA | NA | NA |
| Staekenborg, 2010 (104) | H | Multicenter | VaD | 401 | 73 | 247 | NA | NA | NA | 154 | NA | NA | NA |
| Price, 2009 (105) | H | USA | Controls  Dementia with mild LA  Dementia with moderate LA  Dementia with severe LA | 168 | 78.89 | 8  13  15  5 | NA | NA | NA | 16  60  29  22 | NA | NA | NA |
| Zhou, 2009 (106) | H | China | Controls  MCI from SVD | 136 | 67.06 | 45  36 | NA | NA | NA | 35  20 | NA | NA | NA |
| Gainotti, 2008 (107) | H | Italy | Controls  MCI with multiple SI | 108 | 71.24 | 37  26 | NA | NA | NA | 28  17 | NA | NA | NA |
| Nordlund, 2007 (108) | H | Sweden | Controls  VaMCI | 120 | 66.75 | 28  22 | NA | NA | NA | 32  38 | NA | NA | NA |
| Nordahl, 2005 (109) | H | USA | Controls  MCI with severe WMH | 28 | 78.25 | 3  5 | NA | NA | NA | 14  6 | NA | NA | NA |
| Van Zandvoort, 2005 (110) | H | Netherlands | Controls  LI | 38 | 62.06 | 8  16 | NA | NA | NA | 4  10 | NA | NA | NA |
| Garrett, 2004 (111) | H | USA | Controls  VaCI  VaD | 69 | 77.22 | 11  10  17 | NA | NA | NA | 14  8  9 | NA | NA | NA |
| Graham, 2004 (112) | H | UK | Controls  Subcortical VaD | 38 | 69.65 | 9  14 | NA | NA | NA | 10  5 | NA | NA | NA |
| Van Zandvoort, 2003 (113) | H | Netherlands | LI ^a^ | 17 | 60.3 | 12 | 61.17 | NA | NA | 5 | 58.2 | NA | NA |
| Kramer, 2002 (114) | H | USA | Controls  SIVD | 39 | 73.08 | 12  6 | NA | NA | NA | 15  6 | NA | NA | NA |
| Maeshima, 2002 (115) | H | Japan | Controls  Periventricular WMH | 84 | 48.82 | 32  6 | NA | NA | NA | 38  8 | NA | NA | NA |
| Yuspeh, 2002 (116) | H | USA | Controls  SIVD | 67 | 73.82 | 24  19 | NA | NA | NA | 14  10 | NA | NA | NA |
| Aharon-Peretz, 2000 (117) | H | Israel | VaD | 30 | 71.75 | 20 | NA | NA | NA | 10 | NA | NA | NA |
| Yamauchi, 2000 (118) | H | Japan | Controls  LI | 62 | 68.37 | 13  19 | NA | NA | NA | 21  9 | NA | NA | NA |
| Binetti, 1995 (119) | H | Italy | MID | 32 | 76.1 | 17 | NA | NA | NA | 15 | NA | NA | NA |
| Lewine, 1993 (120) | H | USA | Controls  WMH | 20 | 35.2 | 4  4 | 34.85 | NA | NA | 6  6 | 43.3 | NA | NA |
| Johansson, 2020 (121) | M | Netherlands | Controls  MCI | 157 | 71.0 | 52  31 | NA | NA | NA | 52  22 | NA | NA | NA |
| Atwi, 2018 (122) | M | Canada | Controls  WMH Faz > 2 | 37 | 47.86 | 9  8 | NA | NA | NA | 10  10 | NA | NA | NA |
| Gonçalves, 2017 (123) | M | Portugal | Controls  SVaD | 56 | 75.9 | 19  10 | NA | NA | NA | 21  6 | NA | NA | NA |

Healthy participants included those defined as neurologically, functionally or cognitively healthy or community-dwelling individuals. Total participants refer to the relevant populations extracted for this work. ^a^ Studies that provided more stratified information of SVD groups than controls, so controls were not included in this review. Abbreviations: AIS = acute ischemic stroke, CADASIL = cerebral autosomal dominant arteriopathy with subcortical infarcts and leukoencephalopathy, CMB = cerebral microbleeds, CI = cognitive impairment, DWMH = deep white matter hyperintensities, Faz = Fazecas score, ICH = intracerebral hemorrhage, IS = ischemic stroke, LA = leukoaraiosis, LI = lacunar infarct, LS = lacunar stroke, MCI = mild cognitive impairment, MID = multi-infarct dementia, NA = not available, SI = silent brain infarcts, SIVD = subcortical ischemic vascular dementia, SVD = cerebral small vessel disease, VaCI = vascular cognitive impairment, VaD = vascular dementia, VaMCI = vascular mild cognitive impairment, VWMD = vascular white matter disease, WMH = white matter hyperintensities, WML = white matter lesions.

**Table 2. Results of quantitative tests excluding studies with quality scores < 5.5/8.**

| **Analysis** | **Statistical test** | **Results** |
| --- | --- | --- |
| Trends across study settings: comparisons of sex ratio per study type. | Kruskal-Wallis test, pairwise Mann-Whitney U-tests and Bonferroni post-hoc correction. | Significant differences were in sex ratios across study setting (H = 17.31, df = 2, p < 0.001). Greater sex ratio in HB compared with CB studies (p_corrected_ < 0.001). |
| Correlation between the deviation of the sex ratio and the size of the recruited population. | Spearman’s rank correlation coefficient. | Negative correlation in CB (rho_Spearman_ = -0.44, p = 0.006) and no correlation in HB studies (rho_Spearman_ = 0.028, p = 0.87). |
| Trends across time: comparisons between recent and previously published research. | Mann-Whitney U-test. | No significant differences between sex ratios of recent studies compared with those previously published (U = 715, p = 0.80). This finding was consistent after classifying by study type (U = 230, p = 0.11 in CB; U = 143, p = 0.42 in HB). |
| Trends across SVD severity: Comparisons between healthy to mild SVD and moderate to severe SVD. | Mann-Whitney U-test. | Sex ratio in moderate to severe SVD was greater than in healthy to mild SVD (U = 1,044, p < 0.001). |
| Trends across SVD presentation: Comparisons between healthy to mild covert SVD, moderate to severe covert SVD, cognitive SVD and cerebrovascular SVD. | Kruskal-Wallis test, pairwise Mann-Whitney U-tests and Bonferroni post-hoc correction. | Significant differences in sex ratios across SVD presentation (H = 28.06, df = 3, p < 0.001). Participants with stroke presentations showed a greater sex ratio, when compared with healthy to mild covert SVD (p_corrected_ < 0.001), cognitive SVD (p_corrected_ = 0.002), and moderate to severe covert SVD (p_corrected_ = 0.001). |

Abbreviations: CB = community-based, HB = hospital-based, SVD = cerebral small vessel disease.

**References**

(1) Kim, C.-M., Alvarado, R.L., Stephens, K., Wey, H.-Y., Wang, D.J., Leritz, E.C., and Salat, D.H. (2020). Associations between cerebral blood flow and structural and functional brain imaging measures in individuals with neuropsychologically defined mild cognitive impairment. *Neurobiology of aging* 86**,** 64-74. doi: 10.1016/j.neurobiolaging.2019.10.023.

(2) Cedres, N., Machado, A., Molina, Y., Diaz-Galvan, P., Hernández-Cabrera, J.A., Barroso, J., Westman, E., and Ferreira, D. (2019). Subjective cognitive decline below and above the age of 60: a multivariate study on neuroimaging, cognitive, clinical, and demographic measures. *Journal of Alzheimer's Disease* 68(1)**,** 295-309. doi: 10.3233/JAD-180720.

(3) Dolui, S., Tisdall, D., Vidorreta, M., Jacobs Jr, D.R., Nasrallah, I.M., Bryan, R.N., Wolk, D.A., and Detre, J.A. (2019). Characterizing a perfusion-based periventricular small vessel region of interest. *NeuroImage: Clinical* 23**,** 101897. doi: 10.1016/j.nicl.2019.101897.

(4) Legdeur, N., Visser, P.J., Woodworth, D.C., Muller, M., Fletcher, E., Maillard, P., Scheltens, P., DeCarli, C., Kawas, C.H., and Corrada, M.M. (2019). White matter hyperintensities and hippocampal atrophy in relation to cognition: the 90+ Study. *Journal of the American Geriatrics Society* 67(9)**,** 1827-1834. doi: 10.1111/jgs.15990.

(5) Mishra, A., Chauhan, G., Violleau, M.-H., Vojinovic, D., Jian, X., Bis, J.C., Li, S., Saba, Y., Grenier-Boley, B., and Yang, Q. (2019). Association of variants in HTRA1 and NOTCH3 with MRI-defined extremes of cerebral small vessel disease in older subjects. *Brain* 142(4)**,** 1009-1023. doi: 10.1093/brain/awz024.

(6) Puzo, C., Labriola, C., Sugarman, M.A., Tripodis, Y., Martin, B., Palmisano, J.N., Steinberg, E.G., Stein, T.D., Kowall, N.W., and McKee, A.C. (2019). Independent effects of white matter hyperintensities on cognitive, neuropsychiatric, and functional decline: a longitudinal investigation using the National Alzheimer’s Coordinating Center Uniform Data Set. *Alzheimer's research & therapy* 11(1)**,** 1-13. doi: 10.1186/s13195-019-0521-0.

(7) Staffaroni, A.M., Cobigo, Y., Elahi, F.M., Casaletto, K.B., Walters, S.M., Wolf, A., Lindbergh, C.A., Rosen, H.J., and Kramer, J.H. (2019). A longitudinal characterization of perfusion in the aging brain and associations with cognition and neural structure. *Human brain mapping* 40(12)**,** 3522-3533. doi: 10.1002/hbm.24613.

(8) Tsapanou, A., Habeck, C., Gazes, Y., Razlighi, Q., Sakhardande, J., Stern, Y., and Salthouse, T.A. (2019). Brain biomarkers and cognition across adulthood. *Human brain mapping* 40(13)**,** 3832-3842. doi: 10.1002/hbm.24634.

(9) Yao, H., Mizoguchi, Y., Monji, A., Yakushiji, Y., Takashima, Y., Uchino, A., Yuzuriha, T., and Hashimoto, M. (2019). Low-grade inflammation is associated with apathy indirectly via deep white matter lesions in community-dwelling older adults: The Sefuri study. *International journal of molecular sciences* 20(8)**,** 1905. doi: 10.3390/ijms20081905.

(10) Croall, I.D., Tozer, D.J., Moynihan, B., Khan, U., O’Brien, J.T., Morris, R.G., Cambridge, V.C., Barrick, T.R., Blamire, A.M., and Ford, G.A. (2018). Effect of standard vs intensive blood pressure control on cerebral blood flow in small vessel disease: the preserve randomized clinical trial. *JAMA neurology* 75(6)**,** 720-727. doi: 10.1001/jamaneurol.2017.5153.

(11) Kuriyama, N., Ozaki, E., Mizuno, T., Ihara, M., Mizuno, S., Koyama, T., Matsui, D., Watanabe, I., Akazawa, K., and Takeda, K. (2018). Association between α-Klotho and deep white matter lesions in the brain: a pilot case control study using brain MRI. *Journal of Alzheimer's Disease* 61(1)**,** 145-155. doi: 10.3233/JAD-170466.

(12) Puglisi, V., Bramanti, A., Lanza, G., Cantone, M., Vinciguerra, L., Pennisi, M., Bonanno, L., Pennisi, G., and Bella, R. (2018). Impaired cerebral haemodynamics in vascular depression: insights from transcranial doppler ultrasonography. *Frontiers in psychiatry* 9**,** 316. doi: 10.3389/fpsyt.2018.00316.

(13) Shokouhi, M., Qiu, D., Samman Tahhan, A., Quyyumi, A.A., and Hajjar, I. (2018). Differential associations of diastolic and systolic pressures with cerebral measures in older individuals with mild cognitive impairment. *American journal of hypertension* 31(12)**,** 1268-1277. doi: 10.1093/ajh/hpy104.

(14) Van Rooden, S., van den Berg-Huysmans, A.A., Croll, P.H., Labadie, G., Hayes, J.M., Viviano, R., van der Grond, J., Rombouts, S.A., and Damoiseaux, J.S. (2018). Subjective cognitive decline is associated with greater white matter hyperintensity volume. *Journal of Alzheimer's Disease* 66(3)**,** 1283-1294. doi: 10.3233/JAD-180285.

(15) Bahrani, A.A., Powell, D.K., Yu, G., Johnson, E.S., Jicha, G.A., and Smith, C.D. (2017). White matter hyperintensity associations with cerebral blood flow in elderly subjects stratified by cerebrovascular risk. *Journal of Stroke and Cerebrovascular Diseases* 26(4)**,** 779-786. doi: 10.1016/j.jstrokecerebrovasdis.2016.10.017.

(16) Squarzoni, P., Tamashiro-Duran, J.H., Duran, F.L., Leite, C.C., Wajngarten, M., Scazufca, M., Menezes, P.R., Lotufo, P.A., Alves, T.C., and Busatto, G.F. (2017). High frequency of silent brain infarcts associated with cognitive deficits in an economically disadvantaged population. *Clinics* 72**,** 474-480. doi: 10.6061/clinics/2017(08)04.

(17) Shi, L., Miao, X., Lou, W., Liu, K., Abrigo, J., Wong, A., Chu, W.C., Wang, D., and Mok, V.C. (2017). The spatial associations of cerebral blood flow and spontaneous brain activities with White matter Hyperintensities—An exploratory study using multimodal magnetic resonance imaging. *Frontiers in neurology* 8**,** 593. doi: 10.3389/fneur.2017.00593.

(18) Xu, X., Chan, Q.L., Hilal, S., Goh, W.K., Ikram, M.K., Wong, T.Y., Cheng, C.-Y., Chen, C.L.-H., and Venketasubramanian, N. (2017). Cerebral microbleeds and neuropsychiatric symptoms in an elderly Asian cohort. *Journal of Neurology, Neurosurgery & Psychiatry* 88(1)**,** 7-11. doi: 10.1136/jnnp-2016-313271.

(19) Chung, C.-P., Chou, K.-H., Chen, W.-T., Liu, L.-K., Lee, W.-J., Chen, L.-K., Lin, C.-P., and Wang, P.-N. (2016). Cerebral microbleeds are associated with physical frailty: a community-based study. *Neurobiology of aging* 44**,** 143-150. doi: 10.1016/j.neurobiolaging.2016.04.025.

(20) Promjunyakul, N.-o., Lahna, D.L., Kaye, J.A., Dodge, H.H., Erten-Lyons, D., Rooney, W.D., and Silbert, L.C. (2016). Comparison of cerebral blood flow and structural penumbras in relation to white matter hyperintensities: a multi-modal magnetic resonance imaging study. *Journal of cerebral blood flow & metabolism* 36(9)**,** 1528-1536. doi: 10.1177/0271678X16651268.

(21) Vemuri, P., Lesnick, T.G., Przybelski, S.A., Knopman, D.S., Preboske, G.M., Kantarci, K., Raman, M.R., Machulda, M.M., Mielke, M.M., and Lowe, V.J. (2015). Vascular and amyloid pathologies are independent predictors of cognitive decline in normal elderly. *Brain* 138(3)**,** 761-771. doi: 10.1093/brain/awu393.

(22) Yamawaki, M., Wada‐Isoe, K., Yamamoto, M., Nakashita, S., Uemura, Y., Takahashi, Y., Nakayama, T., and Nakashima, K. (2015). Association of cerebral white matter lesions with cognitive function and mood in J apanese elderly people: a population‐based study. *Brain and behavior* 5(3)**,** e00315. doi: 10.1002/brb3.315.

(23) Annweiler, C., Annweiler, T., Bartha, R., Herrmann, F., Camicioli, R., and Beauchet, O. (2014). Vitamin D and white matter abnormalities in older adults: a cross‐sectional neuroimaging study. *European journal of neurology* 21(12)**,** 1436-e95. doi: 10.1111/ene.12511.

(24) Mortamais, M., Portet, F., Brickman, A.M., Provenzano, F.A., Muraskin, J., Akbaraly, T.N., Berr, C., Touchon, J., Bonafé, A., and Le Bars, E. (2014). Education modulates the impact of white matter lesions on the risk of mild cognitive impairment and dementia. *The American Journal of Geriatric Psychiatry* 22(11)**,** 1336-1345. doi: 10.1016/j.jagp.2013.06.002.

(25) Sarabia-Cobo, C.M., Pérez, V., Hermosilla, C., Nuñez, M.J., and de Lorena, P. (2014). Apathy and leukoaraiosis in mild cognitive impairment and Alzheimer's disease: Multicenter diagnostic criteria according to the latest studies. *Dementia and geriatric cognitive disorders extra* 4(2)**,** 228-235. doi: 10.1159/000363227.

(26) Sims, R., Katzel, L., Lefkowitz, D., Siegel, E., Rosenberger, W., Manukyan, Z., Whitfield, K., and Waldstein, S. (2014). Association of fasting glucose with subclinical cerebrovascular disease in older adults without Type 2 diabetes. *Diabetic medicine* 31(6)**,** 691-698. doi: 10.1111/dme.12385.

(27) Sun, X., Liang, Y., Wang, J., Chen, K., Chen, Y., Zhou, X., Jia, J., and Zhang, Z. (2014). Early frontal structural and functional changes in mild white matter lesions relevant to cognitive decline. *Journal of Alzheimer's Disease* 40(1)**,** 123-134. doi: 10.3233/JAD-131709.

(28) Wiegman, A.F., Meier, I.B., Schupf, N., Manly, J.J., Guzman, V.A., Narkhede, A., Stern, Y., Martinez-Ramirez, S., Viswanathan, A., and Luchsinger, J.A. (2014). Cerebral microbleeds in a multiethnic elderly community: demographic and clinical correlates. *Journal of the neurological sciences* 345(1-2)**,** 125-130. doi: 10.1016/j.jns.2014.07.024.

(29) Farfel, J.M., Nitrini, R., Suemoto, C.K., Grinberg, L.T., Ferretti, R.E.L., Leite, R.E.P., Tampellini, E., Lima, L., Farias, D.S., and Neves, R.C. (2013). Very low levels of education and cognitive reserve: a clinicopathologic study. *Neurology* 81(7)**,** 650-657. doi: 10.1212/WNL.0b013e3182a08f1b.

(30) Minn, Y.-K., Suk, S.-H., Park, H., Cheong, J.-S., Yang, H., Lee, S., Do, S.-Y., and Kang, J.-S. (2013). Tooth loss is associated with brain white matter change and silent infarction among adults without dementia and stroke. *Journal of Korean medical science* 28(6)**,** 929-933. doi: 10.3346/jkms.2013.28.6.929.

(31) Nebes, R.D., Snitz, B.E., Cohen, A.D., Aizenstein, H.J., Saxton, J.A., Halligan, E.M., Mathis, C.A., Price, J.C., Kamboh, M.I., and Weissfeld, L.A. (2013). Cognitive aging in persons with minimal amyloid-β and white matter hyperintensities. *Neuropsychologia* 51(11)**,** 2202-2209. doi: 10.1016/j.neuropsychologia.2013.07.017.

(32) Hernández, M.d.C.V., Booth, T., Murray, C., Gow, A.J., Penke, L., Morris, Z., Maniega, S.M., Royle, N.A., Aribisala, B.S., and Bastin, M.E. (2013). Brain white matter damage in aging and cognitive ability in youth and older age. *Neurobiology of aging* 34(12)**,** 2740-2747. doi: 10.1016/j.neurobiolaging.2013.05.032.

(33) Bartley, M., Bokde, A., Ewers, M., Faluyi, Y., Tobin, W., Snow, A., Connolly, J., Delaney, C., Coughlan, T., and Collins, D. (2012). Subjective memory complaints in community dwelling healthy older people: the influence of brain and psychopathology. *International journal of geriatric psychiatry* 27(8)**,** 836-843. doi: 10.1002/gps.2794.

(34) Salarirad, S., Staff, R.T., Fox, H.C., Deary, I.J., Whalley, L., and Murray, A.D. (2011). Childhood intelligence and brain white matter hyperintensities predict fluid intelligence age 78–81 years: a 1921 Aberdeen birth cohort study. *Age and ageing* 40(5)**,** 562-567. doi: 10.1093/ageing/afr065.

(35) Stewart, R., Godin, O., Crivello, F., Maillard, P., Mazoyer, B., Tzourio, C., and Dufouil, C. (2011). Longitudinal neuroimaging correlates of subjective memory impairment: 4-year prospective community study. *The British Journal of Psychiatry* 198(3)**,** 199-205. doi: 10.1192/bjp.bp.110.078683.

(36) Villeneuve, S., Massoud, F., Bocti, C., Gauthier, S., and Belleville, S. (2011). The nature of episodic memory deficits in MCI with and without vascular burden. *Neuropsychologia* 49(11)**,** 3027-3035. doi: 10.1016/j.neuropsychologia.2011.07.001.

(37) Qiu, C., Cotch, M., Sigurdsson, S., Jonsson, P., Jonsdottir, M., Sveinbjrnsdottir, S., Eiriksdottir, G., Klein, R., Harris, T., and Van Buchem, M. (2010). Cerebral microbleeds, retinopathy, and dementia: the AGES-Reykjavik Study. *Neurology* 75(24)**,** 2221-2228. doi: 10.1212/WNL.0b013e3182020349.

(38) Godin, O., Maillard, P., Crivello, F., Alpérovitch, A., Mazoyer, B., Tzourio, C., and Dufouil, C. (2009). Association of white-matter lesions with brain atrophy markers: the three-city Dijon MRI study. *Cerebrovascular diseases* 28(2)**,** 177-184. doi: 10.1159/000226117.

(39) Anderson, J.F., Saling, M.M., Srikanth, V.K., Thrift, A.G., and Donnan, G.A. (2008). Individuals with first‐ever clinical presentation of a lacunar infarction syndrome: Is there an increased likelihood of developing mild cognitive impairment in the first 12 months after stroke? *Journal of neuropsychology* 2(2)**,** 373-385. doi: 10.1348/174866408x288846.

(40) Miranda, B., Madureira, S., Verdelho, A., Ferro, J., Pantoni, L., Salvadori, E., Chabriat, H., Erkinjuntti, T., Fazekas, F., and Hennerici, M. (2008). Self-perceived memory impairment and cognitive performance in an elderly independent population with age-related white matter changes. *Journal of Neurology, Neurosurgery & Psychiatry* 79(8)**,** 869-873. doi: 10.1136/jnnp.2007.131078.

(41) Christensen, H., Anstey, K.J., Parslow, R.A., Maller, J., Mackinnon, A., and Sachdev, P. (2007). The brain reserve hypothesis, brain atrophy and aging. *Gerontology* 53(2)**,** 82-95. doi: 10.1159/000096482.

(42) Schretlen, D., Inscore, A., Vannorsdall, T., Kraut, M., Pearlson, G., Gordon, B., and Jinnah, H. (2007). Serum uric acid and brain ischemia in normal elderly adults. *Neurology* 69(14)**,** 1418-1423. doi: 10.1212/01.wnl.0000277468.10236.f1.

(43) Au, R., Massaro, J.M., Wolf, P.A., Young, M.E., Beiser, A., Seshadri, S., D’Agostino, R.B., and DeCarli, C. (2006). Association of white matter hyperintensity volume with decreased cognitive functioning: the Framingham Heart Study. *Archives of neurology* 63(2)**,** 246-250. doi: 10.1001/archneur.63.2.246.

(44) Elkins, J., Longstreth, W., Manolio, T., Newman, A., Bhadelia, R., and Johnston, S. (2006). Education and the cognitive decline associated with MRI-defined brain infarct. *Neurology* 67(3)**,** 435-440. doi: 10.1212/01.wnl.0000228246.89109.98.

(45) Wright, C.B., Paik, M.C., Brown, T.R., Stabler, S.P., Allen, R.H., Sacco, R.L., and DeCarli, C. (2005). Total homocysteine is associated with white matter hyperintensity volume: the Northern Manhattan Study. *Stroke* 36(6)**,** 1207-1211. doi: 10.1161/01.STR.0000165923.02318.22.

(46) Deary, I.J., Leaper, S.A., Murray, A.D., Staff, R.T., and Whalley, L.J. (2003). Cerebral white matter abnormalities and lifetime cognitive change: a 67-year follow-up of the Scottish Mental Survey of 1932. *Psychology and aging* 18(1)**,** 140. doi: 10.1037/0882-7974.18.1.140.

(47) Dufouil, C., Alperovitch, A., and Tzourio, C. (2003). Influence of education on the relationship between white matter lesions and cognition. *Neurology* 60(5)**,** 831-836. doi: 10.1212/01.wnl.0000049456.33231.96.

(48) Tsukishima, E., Saito, H., Shido, K., Kobashi, G., Ying-Yan, G., Kishi, R., Niino, M., Kondo, K., and Sugimura, I. (2001). Long-term blood pressure variability and cerebrovascular changes on ct in a community-based elderly population. *Journal of epidemiology* 11(4)**,** 190-198. doi: 10.2188/jea.11.190.

(49) de Groot, J.C., de Leeuw, F.-E., Oudkerk, M., Hofman, A., Jolles, J., and Breteler, M.M. (2000). Cerebral white matter lesions and depressive symptoms in elderly adults. *Archives of general psychiatry* 57(11)**,** 1071-1076. doi: 10.1001/archpsyc.57.11.1071.

(50) Liao, D., Cooper, L., Cai, J., Toole, J., Bryan, N., Burke, G., Shahar, E., Nieto, J., Mosley, T., and Heiss, G. (1997). The prevalence and severity of white matter lesions, their relationship with age, ethnicity, gender, and cardiovascular disease risk factors: the ARIC Study. *Neuroepidemiology* 16(3)**,** 149-162. doi: 10.1159/000368814.

(51) Boone, K.B., Miller, B.L., Lesser, I.M., Mehringer, C.M., Hill-Gutierrez, E., Goldberg, M.A., and Berman, N.G. (1992). Neuropsychological correlates of white-matter lesions in healthy elderly subjects: a threshold effect. *Archives of Neurology* 49(5)**,** 549-554. doi: 10.1001/archneur.1992.00530290141024.

(52) Tupler, L.A., Coffey, C.E., Logue, P.E., Djang, W.T., and Fagan, S.M. (1992). Neuropsychological importance of subcortical white matter hyperintensity. *Archives of Neurology* 49(12)**,** 1248-1252. doi: 10.1001/archneur.1992.00530360046016.

(53) Rao, S.M., Mittenberg, W., Bernardin, L., Haughton, V., and Leo, G.J. (1989). Neuropsychological test findings in subjects with leukoaraiosis. *Archives of Neurology* 46(1)**,** 40-44. doi: 10.1001/archneur.1989.00520370042017.

(54) Jin, H., Ding, Z., Lian, S., Zhao, Y., He, S., Zhou, L., Zhuoga, C., Wang, H., Xu, J., and Du, A. (2020). Prevalence and risk factors of white matter lesions in Tibetan patients without acute stroke. *Stroke* 51(1)**,** 149-153. doi: 10.1161/STROKEAHA.119.027115.

(55) Zhou, Y.-N., Gao, H.-Y., Zhao, F.-F., Liang, Y.-C., Gao, Y., Liu, X.-H., Wang, T., Wang, Z.-G., and Wu, Q.-J. (2020). The study on analysis of risk factors for severity of white matter lesions and its correlation with cerebral microbleeds in the elderly with lacunar infarction. *Medicine* 99(4). doi: 10.1097/MD.0000000000018865.

(56) Jokumsen-Cabral, A., Aires, A., Ferreira, S., Azevedo, E., and Castro, P. (2019). Primary involvement of neurovascular coupling in cerebral autosomal-dominant arteriopathy with subcortical infarcts and leukoencephalopathy. *Journal of neurology* 266(7)**,** 1782-1788. doi: 10.1007/s00415-019-09331-y.

(57) Kate, M., Gioia, L., Jeerakathil, T., Hill, M.D., Gould, B., McCourt, R., Dowlatshahi, D., Coutts, S., Kosior, J., and Demchuk, A. (2019). Aggressive blood pressure reduction is not associated with decreased perfusion in leukoaraiosis regions in acute intracerebral hemorrhage patients. *Plos one* 14(3)**,** e0213645. doi: 10.1371/journal.pone.0213645.

(58) Liang, Y., Chen, Y.-K., Liu, Y.-L., Mok, V.C., Ungvari, G.S., Chu, W.C., Tang, W.-K., Kim, J.S., and Kim, J.-M. (2019). Exploring causal pathways linking cerebral small vessel diseases burden to poststroke depressive symptoms with structural equation model analysis. *Journal of affective disorders* 253**,** 218-223. doi: 10.1016/j.jad.2019.04.092.

(59) Liang, C., Zhang, J., Liu, H., Ma, J., An, Z., Xia, W., and Zhang, X. (2019). Association of COL4A2 Gene Polymorphisms with Lacunar Stroke in Xinjiang Han Populations. *Journal of Molecular Neuroscience* 69(1)**,** 133-139. doi: 10.1007/s12031-019-01342-8.

(60) Ling, Y., De Guio, F., Jouvent, E., Duering, M., Hervé, D., Guichard, J.P., Godin, O., Dichgans, M., and Chabriat, H. (2019). Clinical correlates of longitudinal MRI changes in CADASIL. *Journal of Cerebral Blood Flow & Metabolism* 39(7)**,** 1299-1305. doi: 10.1177/0271678X18757875.

(61) Liu, X., Chen, L., Cheng, R., Luo, T., Lv, F., Fang, W., Gong, J., and Jiang, P. (2019). Altered functional connectivity in patients with subcortical ischemic vascular disease: a resting-state fMRI study. *Brain research* 1715**,** 126-133. doi: 10.1016/j.brainres.2019.03.022.

(62) Liu, G., Tan, X., Dang, C., Tan, S., Xing, S., Huang, N., Peng, K., Xie, C., Tang, X., and Zeng, J. (2019). Regional shape abnormalities in thalamus and verbal memory impairment after subcortical infarction. *Neurorehabilitation and neural repair* 33(6)**,** 476-485. doi: 10.1177/1545968319846121.

(63) Liu, R., Wu, W., Ye, Q., Gu, Y., Zou, J., Chen, X., Jiang, Y., Bai, F., Xu, Y., and Wang, C. (2019). Distinctive and pervasive alterations of functional brain networks in cerebral small vessel disease with and without cognitive impairment. *Dementia and geriatric cognitive disorders* 47(1-2)**,** 55-67. doi: 10.1159/000496455.

(64) Manso-Calderón, R., Cacabelos-Pérez, P., Sevillano-García, M.D., Herrero-Prieto, M.E., and González-Sarmiento, R. (2020). The impact of vascular burden on behavioural and psychological symptoms in older adults with dementia: the BEVASDE study. *Neurological Sciences* 41(1)**,** 165-174. doi: 10.1007/s10072-019-04071-3.

(65) Oudeman, E.A., Greving, J.P., Van den Berg-Vos, R.M., Biessels, G.J., Bron, E.E., van Oostenbrugge, R., de Bresser, J., Kappelle, L.J., and Consortium, H.-B.C. (2019). Nonfocal transient neurological attacks are associated with cerebral small vessel disease. *Stroke* 50(12)**,** 3540-3544. doi: 10.1161/STROKEAHA.119.025328.

(66) Reginold, W., Sam, K., Poublanc, J., Fisher, J., Crawley, A., and Mikulis, D.J. (2019). The efficiency of the brain connectome is associated with cerebrovascular reactivity in persons with white matter hyperintensities. *Human brain mapping* 40(12)**,** 3647-3656. doi: 10.1002/hbm.24622.

(67) Rudilosso, S., Laredo, C., Mancosu, M., Moya-Planas, N., Zhao, Y., Chirife, O., Chamorro, Á., and Urra, X. (2019). Cerebral perfusion and compensatory blood supply in patients with recent small subcortical infarcts. *Journal of Cerebral Blood Flow & Metabolism* 39(7)**,** 1326-1335. doi: 10.1177/0271678X18758548.

(68) Staszewski, J., Skrobowska, E., Piusińska-Macoch, R., Brodacki, B., and Stępień, A. (2019). IL-1α and IL-6 predict vascular events or death in patients with cerebral small vessel disease—Data from the SHEF-CSVD study. *Advances in medical sciences* 64(2)**,** 258-266. doi: 10.1016/j.advms.2019.02.003.

(69) Tsai, H.-H., Pasi, M., Tsai, L.-K., Chen, Y.-F., Chen, Y.-W., Tang, S.-C., Gurol, M.E., Yen, R.-F., and Jeng, J.-S. (2020). Superficial cerebellar microbleeds and cerebral amyloid angiopathy: a magnetic resonance imaging/positron emission tomography study. *Stroke* 51(1)**,** 202-208. doi: 10.1161/STROKEAHA.119.026235.

(70) Wu, X., Ge, X., Du, J., Wang, Y., Sun, Y., Han, X., Ding, W., Cao, M., Xu, Q., and Zhou, Y. (2019). Characterizing the penumbras of white matter hyperintensities and their associations with cognitive function in patients with subcortical vascular mild cognitive impairment. *Frontiers in neurology* 10**,** 348. doi: 10.3389/fneur.2019.00348.

(71) Yu, D., Hennebelle, M., Sahlas, D.J., Ramirez, J., Gao, F., Masellis, M., Cogo-Moreira, H., Swartz, R.H., Herrmann, N., and Chan, P.C. (2019). Soluble epoxide hydrolase-derived linoleic acid oxylipins in serum are associated with periventricular white matter hyperintensities and vascular cognitive impairment. *Translational stroke research* 10(5)**,** 522-533. doi: 10.1007/s12975-018-0672-5.

(72) Zhang, L., Sun, W.-h., Xing, M., Wang, Y., Zhang, Y., Sun, Q., Cheng, Y., Shi, C., and Zhang, N. (2019). Medial temporal lobe atrophy is related to learning strategy changes in amnestic mild cognitive impairment. *Journal of the International Neuropsychological Society* 25(7)**,** 706-717. doi: 10.1017/S1355617719000353.

(73) Ishibashi, M., Kimura, N., Aso, Y., and Matsubara, E. (2018). Effects of white matter lesions on brain perfusion in patients with mild cognitive impairment. *Clinical neurology and neurosurgery* 168**,** 7-11. doi: 10.1016/j.clineuro.2018.02.030.

(74) Kim, H.J., Park, S., Cho, H., Jang, Y.K., San Lee, J., Jang, H., Kim, Y., Kim, K.W., Ryu, Y.H., and Choi, J.Y. (2018). Assessment of extent and role of tau in subcortical vascular cognitive impairment using 18F-AV1451 positron emission tomography imaging. *JAMA neurology* 75(8)**,** 999-1007. doi: 10.1001/jamaneurol.2018.0975.

(75) Lisiecka-Ford, D.M., Tozer, D.J., Morris, R.G., Lawrence, A.J., Barrick, T.R., and Markus, H.S. (2018). Involvement of the reward network is associated with apathy in cerebral small vessel disease. *Journal of affective disorders* 232**,** 116-121. doi: 10.1016/j.jad.2018.02.006.

(76) Anor, C.J., O'Connor, S., Saund, A., Tang-Wai, D.F., Keren, R., and Tartaglia, M.C. (2017). Neuropsychiatric symptoms in Alzheimer disease, vascular dementia, and mixed dementia. *Neurodegenerative Diseases* 17(4-5)**,** 127-134. doi: 10.1159/000455127.

(77) Yuan, J.-L., Wang, S.-K., Guo, X.-J., Teng, L.-l., Jiang, H., Gu, H., and Hu, W.-L. (2017). Disconnections of cortico-subcortical pathways related to cognitive impairment in patients with leukoaraiosis: a preliminary diffusion tensor imaging study. *European neurology* 78(1-2)**,** 41-47. doi: 10.1159/000477899.

(78) Zhong, G., Zhang, R., Jiaerken, Y., Yu, X., Zhou, Y., Liu, C., Lin, L., Tong, L., and Lou, M. (2017). Better correlation of cognitive function to white matter integrity than to blood supply in subjects with leukoaraiosis. *Frontiers in aging neuroscience* 9**,** 185. doi: 10.3389/fnagi.2017.00185.

(79) Bella, R., Cantone, M., Lanza, G., Ferri, R., Vinciguerra, L., Puglisi, V., Pennisi, M., Ricceri, R., Di Lazzaro, V., and Pennisi, G. (2016). Cholinergic circuitry functioning in patients with vascular cognitive impairment–no dementia. *Brain Stimulation* 9(2)**,** 225-233. doi: 10.1016/j.brs.2015.09.013.

(80) Hashimoto, T., Yokota, C., Koshino, K., Shimomura, R., Hino, T., Moriguchi, T., Hori, Y., Uehara, T., Minematsu, K., and Iida, H. (2016). Cerebral blood flow and metabolism associated with cerebral microbleeds in small vessel disease. *Annals of nuclear medicine* 30(7)**,** 494-500. doi: 10.1007/s12149-016-1086-7.

(81) Hsu, Y.-H., Huang, C.-F., Lo, C.-P., Wang, T.-L., Yang, C.-C., and Tu, M.-C. (2016). Frontal assessment battery as a useful tool to differentiate mild cognitive impairment due to subcortical ischemic vascular disease from Alzheimer disease. *Dementia and geriatric cognitive disorders* 42(5-6)**,** 331-341. doi: 10.1159/000452762.

(82) Turk, M., Zaletel, M., and Oblak, J.P. (2016). Characteristics of cerebral hemodynamics in patients with ischemic leukoaraiosis and new ultrasound indices of ischemic leukoaraiosis. *Journal of Stroke and Cerebrovascular Diseases* 25(4)**,** 977-984. doi: 10.1016/j.jstrokecerebrovasdis.2015.12.045.

(83) Brookes, R.L., Hollocks, M.J., Khan, U., Morris, R.G., and Markus, H.S. (2015). The Brief Memory and Executive Test (BMET) for detecting vascular cognitive impairment in small vessel disease: a validation study. *BMC medicine* 13(1)**,** 1-8. doi: 10.1186/s12916-015-0290-y.

(84) Hsu, J.-L., Lee, W.-J., Liao, Y.-C., Lirng, J.-F., Wang, S.-J., and Fuh, J.-L. (2015). Posterior atrophy and medial temporal atrophy scores are associated with different symptoms in patients with Alzheimer’s disease and mild cognitive impairment. *PloS one* 10(9)**,** e0137121. doi: 10.1371/journal.pone.0137121.

(85) Brookes, R.L., Herbert, V., Paul, S., Hannesdottir, K., Markus, H.S., and Morris, R.G. (2014). Executive dysfunction, awareness deficits and quality of life in patients with cerebral small vessel disease: A structural equation model. *Neuropsychology* 28(2)**,** 247. doi: 10.1037/neu0000015.

(86) Delrieu, J., Desmidt, T., Camus, V., Sourdet, S., Boutoleau‐Bretonnière, C., Mullin, E., Vellas, B., Payoux, P., Lebouvier, T., and Initiative, A.s.D.N. (2015). Apathy as a feature of prodromal Alzheimer's disease: an FDG‐PET ADNI study. *International journal of geriatric psychiatry* 30(5)**,** 470-477. doi: 10.1002/gps.4161.

(87) Ledesma-Amaya, L.I., Salvador-Cruz, J., Rodríguez-Agudelo, Y., Valencia-Flores, M., and Arauz, A. (2014). Alteraciones neuropsicológicas asociadas en pacientes con infarto lacunar. *Acta Colombiana de Psicología* 17(2)**,** 43-52. doi: 10.14718/ACP.2014.17.2.5.

(88) Pinkhardt, E.H., Issa, H., Gorges, M., Jürgens, R., Lulé, D., Heimrath, J., Müller, H.-P., Ludolph, A.C., Becker, W., and Kassubek, J. (2014). Do eye movement impairments in patients with small vessel cerebrovascular disease depend on lesion load or on cognitive deficits? A video-oculographic and MRI study. *Journal of neurology* 261(4)**,** 791-803. doi: 10.1007/s00415-014-7275-1.

(89) Zi, W., Duan, D., and Zheng, J. (2014). Cognitive impairments associated with periventricular white matter hyperintensities are mediated by cortical atrophy. *Acta Neurologica Scandinavica* 130(3)**,** 178-187. doi: 10.1111/ane.12262.

(90) Deguchi, K., Kono, S., Deguchi, S., Morimoto, N., Kurata, T., Ikeda, Y., and Abe, K. (2013). A novel useful tool of computerized touch panel–type screening test for evaluating cognitive function of chronic ischemic stroke patients. *Journal of Stroke and Cerebrovascular Diseases* 22(7)**,** e197-e206. doi: 10.1016/j.jstrokecerebrovasdis.2012.11.011.

(91) Fang, M., Feng, C., Xu, Y., Hua, T., Jin, A.-P., and Liu, X.-Y. (2013). Microbleeds and silent brain infarctions are differently associated with cognitive dysfunction in patients with advanced periventricular leukoaraiosis. *International journal of medical sciences* 10(10)**,** 1307. doi: 10.7150/ijms.6430.

(92) Kim, H.J., Kang, S.J., Kim, C., Kim, G.H., Jeon, S., Lee, J.M., Oh, S.J., Kim, J.S., Choe, Y.S., and Lee, K.H. (2013). The effects of small vessel disease and amyloid burden on neuropsychiatric symptoms: a study among patients with subcortical vascular cognitive impairments. *Neurobiology of aging* 34(7)**,** 1913-1920. doi: 10.1016/j.neurobiolaging.2013.01.002.

(93) Narasimhalu, K., Wiryasaputra, L., Sitoh, Y.Y., and Kandiah, N. (2013). Post‐stroke subjective cognitive impairment is associated with acute lacunar infarcts in the basal ganglia. *European Journal of Neurology* 20(3)**,** 547-551. doi: 10.1111/ene.12032.

(94) Sudo, F.K., Alves, C.E.O., Alves, G.S., Ericeira-Valente, L., Tiel, C., Moreira, D.M., Laks, J., and Engelhardt, E. (2013). White matter hyperintensities, executive function and global cognitive performance in vascular mild cognitive impairment. *Arquivos de neuro-psiquiatria* 71**,** 431-436. doi: 10.1590/0004-282X20130057.

(95) van Norden, A.G., van Uden, I.W., de Laat, K.F., Gons, R.A., Kessels, R.P., van Dijk, E.J., and de Leeuw, F.-E. (2013). Cerebral microbleeds are related to subjective cognitive failures: the RUN DMC study. *Neurobiology of aging* 34(9)**,** 2225-2230. doi: 10.1016/j.neurobiolaging.2013.03.021.

(96) Li, C., Ling, X., Liu, S., Xu, A., Zhang, Y., Xing, S., Pei, Z., and Zeng, J. (2012). Abnormalities of magnetic resonance spectroscopy and diffusion tensor imaging are correlated with executive dysfunction in patients with ischemic leukoaraiosis. *Journal of Clinical Neuroscience* 19(5)**,** 718-722. doi: 10.1016/j.jocn.2011.07.052.

(97) Quinque, E.M., Arélin, K., Dukart, J., Roggenhofer, E., Streitbuerger, D.-P., Villringer, A., Frisch, S., Mueller, K., and Schroeter, M.L. (2012). Identifying the neural correlates of executive functions in early cerebral microangiopathy: a combined VBM and DTI study. *Journal of Cerebral Blood Flow & Metabolism* 32(10)**,** 1869-1878. doi: 10.1038/jcbfm.2012.96.

(98) Yi, L., Wang, J., Jia, L., Zhao, Z., Lu, J., Li, K., Jia, J., He, Y., Jiang, C., and Han, Y. (2012). Structural and functional changes in subcortical vascular mild cognitive impairment: a combined voxel-based morphometry and resting-state fMRI study. *PLoS One* 7(9)**,** e44758. doi: 10.1371/journal.pone.0044758.

(99) Fernández, P.J., Campoy, G., Santos, J.M.G., Antequera, M.M., García-Sevilla, J., Castillo, A., Antúnez, C., and Fuentes, L.J. (2011). Is there a specific pattern of attention deficit in mild cognitive impairment with subcortical vascular features? Evidence from the Attention Network Test. *Dementia and Geriatric Cognitive Disorders* 31(4)**,** 268-275. doi: 10.1159/000327165.

(100) Xiong, Y.Y., Wong, A., Mok, V.C., Tang, W.K., Lam, W.W., Kwok, T.C., Chu, W.C., Chan, A.Y., and Wong, L.K. (2011). Frequency and predictors of proxy‐confirmed post‐stroke cognitive complaints in lacunar stroke patients without major depression. *International journal of geriatric psychiatry* 26(11)**,** 1144-1151. doi: 10.1002/gps.2652.

(101) Hassan, M.A., Helmy, S.M., Rabah, A.M., Ameen, A.I., and Helmy, H. (2010). Assessment of patients with lacunar infarction: a magnetic resonance spectroscopic and psychometric study. *Egypt J Neurol Psychiatry Neurosurg* 47(1)**,** 1-10.

(102) Pascual, B., Prieto, E., Arbizu, J., Marti-Climent, J., Olier, J., and Masdeu, J.C. (2010). Brain glucose metabolism in vascular white matter disease with dementia: differentiation from Alzheimer disease. *Stroke* 41(12)**,** 2889-2893. doi: 10.1161/STROKEAHA.110.591552.

(103) Seo, S.W., Ahn, J., Yoon, U., Im, K., Lee, J.M., Tae Kim, S., Ahn, H.J., Chin, J., Jeong, Y., and Na, D.L. (2010). Cortical thinning in vascular mild cognitive impairment and vascular dementia of subcortical type. *Journal of Neuroimaging* 20(1)**,** 37-45. doi: 10.1111/j.1552-6569.2008.00293.x.

(104) Staekenborg, S.S., Su, T., van Straaten, E.C., Lane, R., Scheltens, P., Barkhof, F., and van der Flier, W.M. (2010). Behavioural and psychological symptoms in vascular dementia; differences between small-and large-vessel disease. *Journal of Neurology, Neurosurgery & Psychiatry* 81(5)**,** 547-551. doi: 10.1136/jnnp.2009.187500.

(105) Price, C.C., Garrett, K.D., Jefferson, A.L., Cosentino, S., Tanner, J.J., Penney, D.L., Swenson, R., Giovannetti, T., Bettcher, B.M., and Libon, D.J. (2009). Leukoaraiosis severity and list-learning in dementia. *The Clinical Neuropsychologist* 23(6)**,** 944-961. doi: 10.1080/13854040802681664.

(106) Zhou, A., and Jia, J. (2009). Different cognitive profiles between mild cognitive impairment due to cerebral small vessel disease and mild cognitive impairment of Alzheimer’s disease origin. *Journal of the International Neuropsychological Society* 15(6)**,** 898-905. doi: 10.1017/S1355617709990816.

(107) Gainotti, G., Ferraccioli, M., Vita, M.G., and Marra, C. (2008). Patterns of neuropsychological impairment in MCI patients with small subcortical infarcts or hippocampal atrophy. *Journal of the International Neuropsychological Society* 14(4)**,** 611-619. doi: 10.1017/S1355617708080831.

(108) Nordlund, A., Rolstad, S., Klang, O., Lind, K., Hansen, S., and Wallin, A. (2007). Cognitive profiles of mild cognitive impairment with and without vascular disease. *Neuropsychology* 21(6)**,** 706. doi: 10.1037/0894-4105.21.6.706.

(109) Nordahl, C.W., Ranganath, C., Yonelinas, A.P., DeCarli, C., Reed, B.R., and Jagust, W.J. (2005). Different mechanisms of episodic memory failure in mild cognitive impairment. *Neuropsychologia* 43(11)**,** 1688-1697. doi: 10.1016/j.neuropsychologia.2005.01.003.

(110) van Zandvoort, M.J., Van der Grond, J., Kappelle, L., and De Haan, E. (2005). Cognitive deficits and changes in neurometabolites after a lacunar infarct. *Journal of neurology* 252(2)**,** 183-190. doi: 10.1007/s00415-005-0629-y.

(111) Garrett, K.D., Browndyke, J.N., Whelihan, W., Paul, R.H., DiCarlo, M., Moser, D.J., Cohen, R.A., and Ott, B.R. (2004). The neuropsychological profile of vascular cognitive impairment—no dementia: comparisons to patients at risk for cerebrovascular disease and vascular dementia. *Archives of Clinical Neuropsychology* 19(6)**,** 745-757. doi: 10.1016/j.acn.2003.09.008.

(112) Graham, N., Emery, T., and Hodges, J. (2004). Distinctive cognitive profiles in Alzheimer’s disease and subcortical vascular dementia. *Journal of Neurology, Neurosurgery & Psychiatry* 75(1)**,** 61-71. doi: 10.1016/s0084-3970(08)70286-x.

(113) Van Zandvoort, M., De Haan, E., Van Gijn, J., and Kappelle, L.J. (2003). Cognitive functioning in patients with a small infarct in the brainstem. *Journal of the International Neuropsychological Society* 9(3)**,** 490-494. doi: 10.1017/S1355617703000146.

(114) Kramer, J., Reed, B.R., Mungas, D., Weiner, M., and Chui, H. (2002). Executive dysfunction in subcortical ischaemic vascular disease. *Journal of Neurology, Neurosurgery & Psychiatry* 72(2)**,** 217-220. doi: 10.1136/jnnp.72.2.217.

(115) Maeshima, S., Moriwaki, H., Ozaki, F., Okita, R., Yamaga, H., and Ueyoshi, A. (2002). Silent cerebral infarction and cognitive function in middle‐aged neurologically healthy subjects. *Acta neurologica scandinavica* 105(3)**,** 179-184. doi: 10.1034/j.1600-0404.2002.1o068.x.

(116) Yuspeh, R.L., Vanderploeg, R.D., Crowell, T.A., and Mullan, M. (2002). Differences in executive functioning between Alzheimer’s disease and subcortical ischemic vascular dementia. *Journal of Clinical and Experimental Neuropsychology* 24(6)**,** 745-754. doi: 10.1076/jcen.24.6.745.8399.

(117) Aharon-Peretz, J., Kliot, D., and Tomer, R. (2000). Behavioral differences between white matter lacunar dementia and Alzheimer’s disease: a comparison on the neuropsychiatric inventory. *Dementia and geriatric cognitive disorders* 11(5)**,** 294-298. doi: 10.1159/000017252.

(118) Yamauchi, H., Fukuyama, H., and Shio, H. (2000). Corpus callosum atrophy in patients with leukoaraiosis may indicate global cognitive impairment. *Stroke* 31(7)**,** 1515-1520. doi: 10.1161/01.str.31.7.1515.

(119) Binetti, G., Padovani, A., Magni, E., Bianchetti, A., Scuratti, A., Lenzi, G., and Trabucchi, M. (1995). Delusions and dementia: clinical and CT correlates. *Acta Neurologica Scandinavica* 91(4)**,** 271-275. doi: 10.1111/j.1600-0404.1995.tb07003.x.

(120) Lewine, R., Hudgins, P., Risch, S.C., and Walker, E.F. (1993). Lowered attention capacity in young, medically healthy men with magnetic resonance brain hyperintensity signals. *Neuropsychiatry, Neuropsychology, & Behavioral Neurology*.

(121) Johansson, M., Stomrud, E., Lindberg, O., Westman, E., Johansson, P.M., van Westen, D., Mattsson, N., and Hansson, O. (2020). Apathy and anxiety are early markers of Alzheimer's disease. *Neurobiology of Aging* 85**,** 74-82. doi: 10.1016/j.neurobiolaging.2019.10.008.

(122) Atwi, S., Metcalfe, A.W., Robertson, A.D., Rezmovitz, J., Anderson, N.D., and MacIntosh, B.J. (2018). Attention-related brain activation is altered in older adults with white matter hyperintensities using multi-echo fMRI. *Frontiers in Neuroscience* 12**,** 748. doi: 10.3389/fnins.2018.00748.

(123) Goncalves, C., Pinho, M.S., Cruz, V., Gens, H., Oliveira, F., Pais, J., Rente, J., Santana, I., and Santos, J.M. (2017). Portuguese version of Wechsler Memory Scale–3rd edition’s utility with demented elderly adults. *Applied Neuropsychology: Adult* 24(3)**,** 212-225. doi: 10.1080/23279095.2015.1135440.
